# Toll-like receptor 7/8 agonist stimulation rapidly skews the antibody repertoire of B cells in non-human primates

**DOI:** 10.3389/fimmu.2025.1647209

**Published:** 2025-09-22

**Authors:** Shiyu Wang, Michael Citron, Lihua Luo, I-Ming Wang, Xiao Liu, Wei Zhang

**Affiliations:** ^1^ Department of cell biology and neurology, School of Life Sciences, Xuzhou Medical University, Xuzhou, China; ^2^ Merck & Co. Inc., Kenilworth, NJ, United States; ^3^ BGI-Shenzhen, Shenzhen, China; ^4^ Pfizer Translational Oncology, San Diego, CA, United States; ^5^ Shenzhen International Graduate School, Tsinghua University, Shenzhen, China; ^6^ Department of Biomedical Informatics, School of Life Sciences, Central South University, Changsha, China

**Keywords:** toll-like receptor 7 and 8 agonist, antibody repertoire, adjuvant development, non-human primates, B cells

## Abstract

**Introduction:**

Toll-like receptors 7 and 8(TLR7/8) recognize purine-rich single-stranded RNA from pathogens, triggering immune responses. Although TLR7/8 agonists are emerging as potential novel adjuvants, their impact on the adaptive B cell response, particularly antibody repertoire, remains unclear.

**Methods:**

Six Indian rhesus macaques(IRMs) and six African green monkeys(AGMs) were stimulated with a TLR7/8 agonist. Peripheral blood samples were collected pre-stimulation and multiple timepoints within one week post-stimulation for flow cytometry and antibody repertoire sequencing.

**Results:**

B cell activation was observed at 24 hours. Significant increases in antibody repertoire diversity were detected at 48 and 72 hours in both species; however, diversity remained elevated at one week in IRMs but returned to baseline in AGMs. Analysis of antibody lineage distributions revealed a marked increase in increased lineages at 48 and 72 hours, characterized by higher frequency, a greater number of antibody clonotypes, and increased mutation rates. The identified expanded lineages, induced by the TLR7/8 agonist, exhibited λ chain bias and divergent antibodies across individuals, and primarily originated from highly mutated antibodies, likely corresponding to memory/effector-like B cells.

**Conclusions:**

Our findings demonstrate that the TLR7/8 agonist rapidly induces a divergent expansion of B cells, establishing an immune foundation supporting vaccine responses and offering new insights into the dynamic B cell modulation.

## Introduction

Toll-like receptors (TLRs) are a large family of proteins expressed in various types of immune cells and constitute a class of pattern recognition receptors (PRRs) that recognize molecules shared by pathogens, referred to as pathogen-associated molecular patterns (PAMPs). TLRs have been identified as the great potential of PRR-targeting adjuvants for vaccines to enhance immune responses (1). Both TLR4 and TLR9 agonists have been demonstrated to be effective adjuvants and have been incorporated into multiple vaccines (2–5). Human TLR7 and TLR8 (TLR7/8), which are expressed on the endosomal surface of dendritic cells (DCs) and other immune cells, are phylogenetically similar (6) and are capable of recognizing purine-rich single-stranded RNA (ssRNA) from pathogens to elicit immune responses. In recent years, TLR7 and TLR8 agonists, as relatively novel adjuvants, have been increasingly investigated in both clinical and nonclinical studies (7, 8). Several organizations were developing TLR7 and TLR8 agonists for use as vaccine adjuvants (9). Notably, BBV152, a COVID-19 vaccine developed in India, incorporates a TLR7/8 agonist as an adjuvant (10).

The majority studies on TLR7/8 agonist function have focused on their impact on antigen-presenting cells (APCs), such as DCs and macrophages, demonstrating the improvement of APCs activation and induction of immune-modulatory cytokines (11–13). However, as vaccine adjuvants, their ability to augment the adaptive immune response is particularly critical. There are relatively few studies investigating the impact of TLR7/8 agonist on the adaptive immune response, particularly on B cells, which secrete antibodies. Imiquimod, a TLR 7 agonist, has been shown to directly activate purified mouse peritoneal B cells, and when combined with inactivated influenza virus particle in a vaccine formulation, it facilitates the differentiation of naive B cells into the B cells producing vaccine-induced antibodies (14). In a mouse retroviral model, TLR7 has been identified as one of requirements for an antibody response to infection (15). Additionally, immunization of mice with a combination of a TLR7 agonist and an antigen has been shown to promote memory B cells response to antigens (16). Although the studies suggest that TLR7 agonists contribute to the enhancement of B cells-mediated antibody responses, the dynamic changes in B cell populations and the antibody repertoire remain poorly understood. Since the induction of effective memory B cells and IgG antibodies is a key objective of vaccines, a deeper understanding of antibody response induced by potential adjuvant is crucial for vaccine design.

Antibody repertoire, also known as B-cell receptor repertoire, is highly diverse in humans, estimated to encompass over 10 (13) potential clonotypes (17). Empirical measurements indicate that the B-cell compartment comprises approximately 1.8 × 10^12^ cells in real sample (18) This diversity arises from both V(D)J rearrangement during B cell development and somatic hypermutation (SHM) or antibody isotype switching during B cell maturation. Traditional methods have been unable to fully capture this vast diversity until the advent of high-throughput sequencing technologies. In our previously studies, we developed antibody sequencing technologies, including experimental methodologies for capturing the diverse antibody repertoire, followed by next generation sequencing (19–23), and also developed corresponding bioinformatical tools for data analysis (24–26). Antibody sequencing enables the monitoring of dynamic changes in the antibody repertoire on before and after vaccination, including the characterization of vaccine-induced antibody expansion, SHM, and isotype class switching (21, 27–29). For preclinical vaccine studies, non-human primates, such as monkeys, are commonly used as models for vaccine evaluation and testing, as their immune systems closely resemble those of humans (30). We previously developed an antibody sequencing method for rhesus macaque and applied it to systematically evaluate adaptive immune response for multiple preclinical human immunodeficiency virus (HIV) vaccines (21, 23).

In this study, we assessed the impact of TLR7/8 agonists on adaptive immune responses, providing insights into their potential as vaccine adjuvants. Both Indian rhesus macaques (IRMs) and African green monkeys (AGMs) were stimulated with a TLR7/8 agonist, and peripheral blood samples were collected from pre-stimulation and multiple timepoints within one week post-stimulation. The antibody repertoire was sequenced and skewing patterns in repertoire characteristics were analyzed in depth.

## Materials and methods

### Animals, injections, and sample collection

Six disease-free rhesus macaques (*Macaca mulatta*) and six African green monkeys (*Chlorocebus aethiops*) were used in this study. Detailed records of these non-human primates (NHPs) are provided in **Supplementary Table S1**. Each animal received an injection of 2.5 mg of IDR-053, a synthetic TLR7/8 agonist with the sequence 5’-X5UGCUGCUUGUG-X-GUGUUCGUCGUX5-5’, where X is a glycerol linker and X5 is 1,5-pentanediol. The synthesis protocol for this molecule is described in patent EP2357231A2. All animals were heathy and fully conscious before and after the injection. They were housed in accordance with the standards of the Guide for the Care and Use of Laboratory Animals, both during and after the experiment period. The study was approved by the Merck Institutional Animal Care and Use Committee (IACUC) and the Institutional Review Board (IRB) of BGI-Shenzhen (No. 13040).

Peripheral blood was collected prior to stimulation to establish a baseline. Following the administration of 25 mg of IDR-053, blood samples were collected at 8, 24, 48, and 72 hours, as well as 1-week post-stimulation. Approximately 2.6 mL of blood collected at 0, 24, 72 hours, and 1 week was analyzed by flow cytometry.

### Flow cytometry

For cell surface staining, fresh PBMCs were resuspended in 100 μL of flow cytometry staining buffer (3% bovine serum albumin in phosphate-buffered saline) containing primary antibody ligated with biotin against CD3 (**Supplementary Table S2**), incubated for 40 min at 4 °C and were washed with FACS staining buffer. Cells were then resuspended in 100 μL of flow cytometry staining buffer containing antibodies against biotin, CD20, and HLA-DR (**Supplementary Table S2**) and were incubated for 40 min at 4 °C. For intracellular staining, cells were washed with flow cytometry staining buffer, permeabilized using the Cytofix/Cytoperm Kit (BD Biosciences, Cat. no: 554714), and then stained with anti-Ki-67 antibody. The stained cells were analyzed by a BD FACS Aria II cell sorter (BD Biosciences). B cells were gated by CD3^-^/CD20^low/+^ (**Supplementary Figure S1**).

### Library preparation and sequencing

Peripheral blood mononuclear cells (PBMCs) were isolated from blood using Ficoll-Paque (GE Healthcare) centrifugation method. Total RNA was extracted from PBMCs using Trizol (Invitrogen, 15596-026). For each sample, 600 ng of RNA was used for antibody repertoire library preparation. We performed 5’-Rapid amplification of complementary DNA ends (5’RACE, Invitrogen, 18374-058) method with single-end-specific primers to specifically amplify the BCR repertoire, and then the PCR products were fragmented into 150–200 bp using Covaris E220, which described in details in our previous studies (21, 31). Primers targeting the constant regions of both heavy chain (21) and light chain (**Supplementary Table S3**) were used. The sequencing library were quantified using Agilent 2100 Bioanalyzer system, and then were sequenced via Hiseq2000 (Illumina) using paired-end 150 bp.

### Antibody repertoire sequencing data analysis

Raw data were processed using IMonitor (24), as described previously. Briefly, 1) low-quality reads were filtered out, and paired-end reads were merged; 2) Merged sequences were aligned to the IMGT (http://www.imgt.org, for IgKL) or KIMDB database (32) (for IgH) reference sequences using BLAST, and followed by a re-alignment step to improve the accuracy of alignment, and then V and J genes were assigned for each sequence; 3) Complementarity-determining region 3 (CDR3) region was determined based on assigned V and J genes. Sequencing errors were corrected by sequencing qualities and frequencies. Clonotypes were defined as sequences with the same V and J gene usage and identical CDR3 animo-acid sequences. The statistics of processed data are shown (**Supplementary Table S4**).

### BCR lineages analysis

Lineages were defined as antibody sequences sharing the same V- and J-gene and differing by no more than one amino-acid mismatch within the CDR3; thus the similar clonotypes were clustered into a single lineage, indicating derivation from a common ancestral B cell (33). Lineages were identified separately for IgH and IgKL. Data from all timepoints of the same monkey are combined for lineage identification.

### Diversity and clonality evaluation

We randomly subsampled 500,000 light-chain and 400,000 heavy-chain sequences per sample for diversity estimation. There are several indices as follow.

Shannon index: an overall diversity of BCR repertoire.


$$Shannon\;index=\;-\sum_{i=1}^{n}p_{i}\text{ln}\;p_{i}$$

where *p_i_* is the frequency of the *i* th clonotype in a sample, and *n* is the total number of clonotypes. For the calculation of lineage diversity, lineages were used in place of clonotypes.

Gini index: an overall diversity of BCR repertoire.


$$Gini\;index=\;\frac{\sum_{i=1}^{n}\sum_{j=1}^{n}\left|x_{i}-x_{j}\right|}{2\sum_{i=1}^{n}\sum_{j=1}^{n}x_{j}}$$

where $x_{i}\;$ and $x_{j}\;$ denote the frequency of the *i*th and *j*th gene, clonotype or lineage.

Top 100: it represents the cumulative frequency of the top100 clonotypes or lineages.


$$Top100=\;\sum_{i=1}^{100}p_{i}$$

where ​*p_i_* is the frequency of clonotype *i*, sorted in descending order by clonotype frequency. For the calculation of lineage Top 100, lineages were used in place of clonotypes.

### Morisita–Horn similarity index

For two samples A and B, the Morisita–Horn similarity index (CH) is calculated as:


$$C_{H}=\frac{2\sum_{i=1}^{S}x_{i}y_{i}}{\left(\frac{\sum_{i=1}^{S}x_{i}^{2}}{X^{2}}+\frac{\sum_{i=1}^{S}y_{i}^{2}}{Y^{2}}\right)XY}$$

where *x_i_* and *y_i_* is the counts of *i*th CDR3 in samples A and B, respectively, *X* and *Y* are the total CDR3 counts in A and B, and *S* is the number of unique CDR3 sequences.

### Mutation rate calculation

Based on sequence alignments, mismatches in the V and J regions were counted for each antibody sequence, separately for IgH and IgKL. The somatic hypermutation rate was calculated as:


$$SHM\left(\%\right)=\frac{\sum_{i=1}^{n}v_{i}+\sum_{i=1}^{n}j_{i}}{\sum_{i=1}^{n}V_{i}+\sum_{i=1}^{n}J_{i}}*100$$

where *v_i_* and *j_i_* are the numbers of mismatches in the V and J regions of sequence *i*, and *V_i_* and *J_i_* are the corresponding region lengths.

### Increased lineages and decreased lineages identification

Lineages were assigned per monkey across all six timepoints; consequently, each lineage may contain IgH or IgK/IGL clonotypes sampled at multiple timepoints. For each lineage, clonotypes were first grouped by timepoint, and then three indices were calculated for each timepoint: cumulative frequency, number of unique CDR3 sequences, and mean mutation rate. For each index we computed the fold change (FC) relative to the pre-stimulation baseline (0 hr), and took the base-2 logarithm:


$$log_{2}\left(FC\right)=log_{2}\frac{index_{post-stimulation}}{index_{pre-stimulation}}$$

A log2​(FC)>0 indicates an increase relative to baseline. We defined a lineage as increased lineage for a given index when log2​(FC)>1 (i.e., ≥2-fold increase), and as decreased lineage when log2​(FC)<0.5. Increased and decreased lineages were identified independently for each of the three indices; thus, a given lineage could be classified as increased by one metric (e.g., cumulative frequency) but decreased by another (e.g., mutation rate).

### TLR7/8 agonist-induced expanded lineages identification

We observed that the number of increased lineages was significantly higher at 48hr and 72hr post-stimulation compared with decreased lineages, indicating that TLR7/8 agonist–induced lineages were present at these timepoints. From the pool of increased lineages, a lineage was classified as a TLR7/8 agonist–induced expanded lineage if its log2​(FC) at either 48hr or 72hr met the following criteria: (1) IgKL: frequency log2​(FC) > 3 and absolute frequency > 0.001%; unique CDR3 number log2​(FC) > 2 and unique CDR3 number > 5; mutation rate log2​(FC) > 1.3 and mutation rate > 6%. (2) IgH: frequency log2​(FC) > 3 and frequency > 0.001%;mutation rate log2​(FC) > 1.5 and mutation rate > 5%.

### Phylogenetic tree construction for expanded lineages

PCR products yielded antibody sequences with variable V-region lengths (ranging from 10 bp to 160 bp). To obtain uniform-length sequences for tree construction, we extracted from each antibody sequence a subsequence comprising a 10-bp non-CDR3 V-region segment, the CDR3, and the J-gene region. Based on our lineage definition, all subsequences within a lineage were of equal length; therefore, multiple sequence alignment was not performed. Using these subsequences as input, phylogenetic trees were constructed by the Neighbor-Joining method implemented in MEGA7 (34).

### Statistics

Statistical comparisons between paired groups were performed using the Wilcoxon signed-rank test. When multiple tests were performed within a single figure, p-values were adjusted for false discovery rate (FDR), with adjusted P< 0.05 considered significant. Correlations were assessed using Spearman’s rank correlation coefficient. All analyses were conducted using in-house scripts in Perl (v5.26.2) and R (v3.6.0), employing the ggplot2 and dplyr packages.

### Data availability

The raw sequencing data are available at the China National GeneBank DataBase (CNGBdb) under accession number CNP0000866.

## Results

### TLR7/8 agonist activates T and B lymphocytes 24 hours post-stimulation

**Figure 1:**
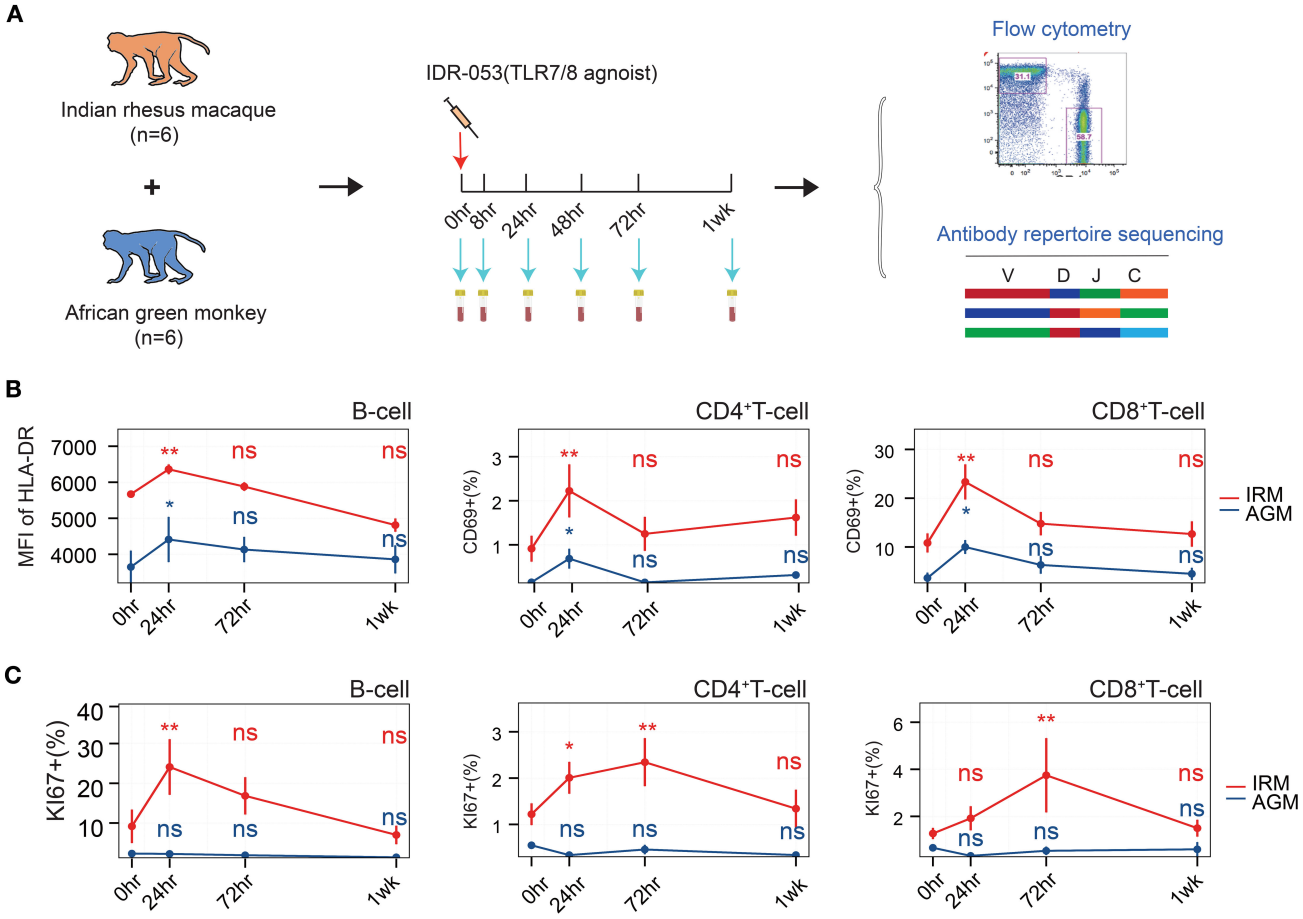
Flowchart of study design and lymphocyte assessment via flow cytometry. **(A)** Flowchart of study design. **(B)** Mean fluorescence intensity (MFI) of HLA-DR expression on B cells (left), frequency of CD69^+^ CD4^+^ T cells (middle), and frequency of CD69^+^ CD8^+^ T cells (right), as measured by flow cytometry. **(C)** Frequency of Ki67^+^ B cells (left, within total B cells), Ki67^+^ CD4^+^ T cells (median, within total CD4^+^ T cells), and Ki67^+^ CD8^+^ T cells (right, within total CD8^+^ T cells), as assessed by flow cytometry. (Paired Wilcox-ranked test, vs. 0hr, *p< 0.05, **p< 0.01). ns, not significant.

To investigate the B cell immune response, particularly the antibody repertoire, under a TLR7/8 agonist stimulation, six IRMs and six AGMs were included and each individual received an injection of TLR7/8 agonist (**Figure 1A**). Peripheral blood samples were collected at baseline (pre-stimulation) and at 8, 24, 48, and 72 hours (hr), as well as one week (1wk) post-stimulation. These samples were used to assess lymphocyte population via flow cytometry (**Supplementary Figure S1**) and to monitor dynamic changes in the antibody repertoire through antibody sequencing. The counts of B cells, CD4+ T cells, and CD8+ T cells in peripheral blood transiently decreased at 24hr post-stimulation in both IRMs and AGMs (**Supplementary Figure S2**), consistent with previous studies (35–37). This suggests the migration of these immune cells to lymphoid tissues, transient activation, and redistribution. To further evaluate lymphocyte activation, we measured HLA-DR expressed on B cells and CD69 expressed on T cells using flow cytometry. The mean fluorescence intensity (MFI) of HLA-DR on B cells increased at 24hr, while CD69 expression on CD4^+^ and CD8^+^ T cells was also upregulated at this timepoint (**Figure 1B**), indicating activation of both B and T cells. Furthermore, the cell proliferation marker Ki67 was examined in both B and T cells. Ki67^+^ B cells and Ki67^+^ CD4^+^ T cells increased at 24hr, and Ki67^+^ CD4^+^ and CD8^+^ T cells increased at 72hr in IRMs, whereas Ki67 expression did not change in AGMs (**Figure 1C**), suggesting the proliferation of lymphocytes in IRMs but not in AGMs. Taken together, the findings indicate that the TLR7/8 agonist induces lymphocyte redistribution at 24hr, accompanied by B and T cell activation and proliferation.

### TLR7/8 agonist skews gene usage and increases antibody repertoire diversity from 48 hours post-stimulation

**Figure 2:**
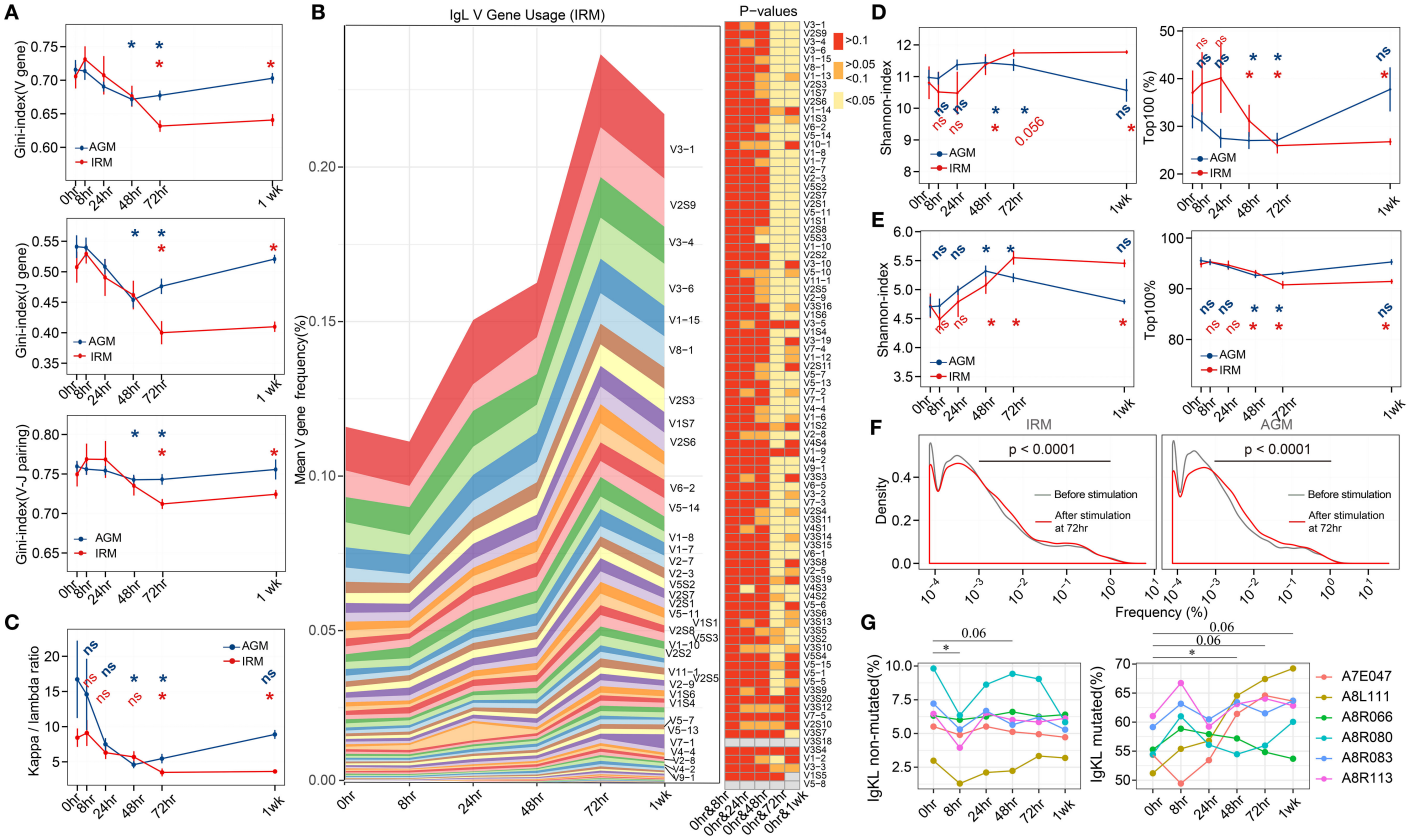
IgKL V/J gene usage and antibody repertoire diversity following TLR7/8 agonist stimulation. **(A)** Gini-index of V genes (left), J genes (middle), and V-J genes pairings(right). **(B)** Mean frequencies of IgL V-gene usage in IRM across all timepoints. Heatmaps display p-values for comparisons of V-gene usage between 0hr and each subsequent timepoint. The left panel shows that the V genes were significantly different (p< 0.05) at both 72 hours and 1 week (last seven genes omitted for space). **(C)** κ/λ ratio of the IgKL repertoire. **(D)** Shannon index (left) and the cumulative frequency of top 100 clonotypes (right). **(E)** Shannon index (left) and the cumulative frequency of top 100 lineages. **(F)** Frequency distribution of lineages presented at both 0hr (grey) and 72hr (red), assessed using the Kolmogorov–Smirnov test. **(G)** The proportion of IgKL non-mutated (mutation rates<0.5%) sequences and mutated (mutation rates >4%) sequences. (Paired Wilcox-ranked test, *p< 0.05). ns, not significant.

To evaluate the specific change of B cells induced by TLR7/8 agonist stimulation, the antibody repertoire, including immunoglobulin heavy-chain (IgH) and kappa and lambda light-chain (IgKL), was obtained by antibody sequencing. Due to the lack of identified germline genes in AGMs, IgH repertoire of AGMs were not presented. The gene usage and diversity of the antibody repertoire were first examined to provide a global overview. The Gini-index of IgKL V genes, J genes, and V-J gene pairings decreased markedly from 72hr to 1wk in IRMs, while in AGMs, it declined at both 48hr and 72hr but returning to baseline at 1wk (**Figure 2A**), suggesting an increase in gene-level diversity. We then illustrated the frequency change of each V gene, separated into IgK and IgL, and found that the abundance of IgL V genes increased after stimulation, with the most significantly expanded genes observed from λ chain in both IRM and AGM (**Figure 2B**; **Supplementary Figure S3, S4**), which was further confirmed by κ/λ ratio analyses (**Figure 2C**). The trends of the κ/λ ratio in both IRMs and AGMs were consistent with changes in gene diversity, indicating an increased frequency of λ genes usage following TLR7/8 agonist stimulation. To validate this, we used TRUST4 (38) to analyze RNA-seq data from B cells cultured with a TLR7/8 agonist (R848) (39). In two of three donors, we observed a decrease in the κ:λ ratio (**Supplementary Figure S5**).

For clonotype repertoire diversity, Shannon-index increased from 48hr to 1wk in IRMs, while in AGMs, it rose at 48hr and 72hr but declined at 1wk in AGMs (**Figure 2D**). However, the cumulative frequency of the top100 clonotypes showed the opposite trends (**Figure 2D**), indicating that increased diversity was driven by a more balanced distribution of clonotypes rather than the very high-frequency ones. The diversities were further confirmed using the tool Recon (40), which calculates both observed and estimated diversity metrics (**Supplementary Figure S6**). Since antibodies undergo proliferation, SHM, and isotype class switching for affinity maturation, lineages—representing functional units—were defined by clustering the similar antibodies derived from the same original B cell source (21). We then examined the overall diversity indices as well as the top100 lineages and found their kinetic changes following stimulation mirrored those observed in clonotypes (**Figure 2E**). To further investigate the frequency distribution of lineages contributing to the increased diversity, we compared lineage frequency distributions at 72hr to those at pre-stimulation. We found that the percentage of lineages with a frequency<0.001% decreased, while those with a frequency between 0.001% and 1% increase in both IRMs and AGMs (**Figure 2F**), suggesting that lineages with moderate frequency contributed to the observed diversity increase. Since SHM contributes to affinity maturation, we examined the mutation rate distribution for all samples in IRM. Almost all samples exhibited three mutation peaks (**Supplementary Figure S7**), likely corresponding to naïve cells with very low mutation rates (IgKL non-mutated, mutation<0.5%), memory/effector cells with high mutation rates (IgKL mutated, mutation>4%), and mixed cell types with intermediate mutation rates. The proportion of IgKL non-mutated sequences decreased at 8hr, whereas IgKL mutated sequences increased at 48hr and maintained this trend in five individuals (**Figure 2G**).

For the IgH repertoire in IRM, V-gene usage was relatively stable, although several V genes showed significant changes from 48hr to 1wk (**Supplementary Figure S8**). For clonotype- and lineage-based diversity, the Shannon index and Top100 metrics did not differ between timepoints; however, Recon-estimated diversity decreased at both 48hr and 72hr (**Supplementary Figure S9**). Mutation rate distributions also exhibited three peaks across samples (**Supplementary Figure S10**). IgM and IgD sequences displayed low mutation rates (<0.5%), whereas IgA, IgG, and IgE sequences showed high mutation rates (>3%) (**Supplementary Figure S11**). The proportion of IgG decreased slightly at 24hr, whereas the proportions of other isotypes remained stable across timepoints (**Supplementary Figure S12A**). This decline in IgG may correspond to the reduction in absolute B-cell counts at the same time point (**Supplementary Figure S2**). Consistent with single-cell sequencing studies reporting that naïve B cells primarily express IgM or IgD with very low mutation rates, while memory and effector B cells mainly express IgA or IgG with high mutation rates (41), we partitioned sequences into IgM/D non-mutated (mutation rate< 0.5%) and IgA/G/E mutated (mutation rate > 3%) groups as proxies for naïve and memory/effector cells, respectively. Using this classification, IgM/D non-mutated sequences decreased at 48hr, whereas IgA/G/E mutated sequences increased at 48hr (**Supplementary Figure S12B**).

### TLR7/8 stimulation induces significant antibody lineage expansion through multiple mechanisms

**Figure 3:**
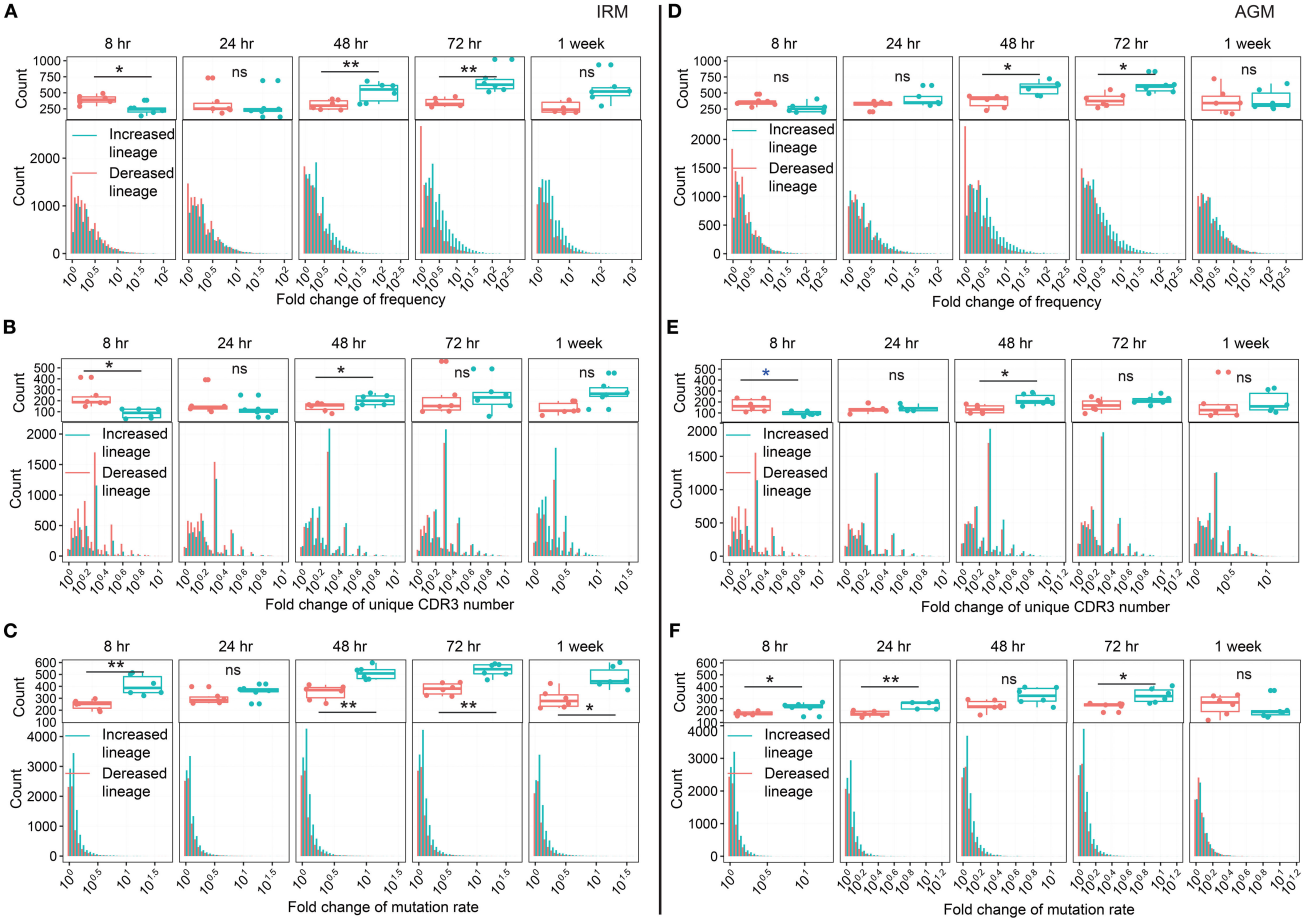
Fold change in three characteristics of increased and decreased lineage distribution in the IgKL chain following TLR7/8 stimulation. **(A, D)** Fold change (FC) in frequency of increased and decreased lineage in IRMs **(A)** and AGMs **(D)**. **(B, E)** FC in the number of unique CDR3 sequences for increased and decreased lineage in IRMs **(B)** and AGMs **(E)**. **(C, F)** FC in mutation rate for increased and decreased lineage in IRMs **(D)** and AGMs **(F)**. The upper plots in **(A, D)** indicate the number of lineages with a frequency FC > 3. The upper plots in **(B, E)** indicate the number of lineages with a unique CDR3 number FC > 2. The upper plot of **(C, F)** indicate the number of lineages with a mutation rate FC > 1.3. (Paired Wilcox-ranked test, vs. 0hr, *p< 0.05, **p< 0.01). ns, not significant.

Since we observed an increase in the diversity of antibody lineages, we further examined their characteristics. The definition of lineage reflects the affinity maturation process of B cells, which involves cell proliferation and SHM. These processes could correspond to the three characteristic analyses of lineage: frequency, unique CDR3 count, and mutation rate. At each timepoint of post-stimulation, lineages were classified as either increased or decreased by comparing the fold change (FC) of each characteristic with those at 0hr (see Methods). Under normal physiological conditions, without external interventions, the distributions of increased and decreased lineages would theoretically remain the same to maintain the repertoire homeostasis. However, external stimuli such as TLR7/8 stimulation may disrupt this equilibrium. First, we directly compared the distributions of the three characteristics between increased and decreased lineages. We found that lineages with either a frequency > 0.001% or a unique CDR3 count > 5 were significantly more prevalent among the increased lineages than among the decreased lineages from 48hr to 1wk post-stimulation, while the lineages with a mutation rate > 6% among the increased lineages were notably more frequent from 8hr to 1wk post-stimulation in both IRMs and AGMs (**Supplementary Figure S13**). We further calculated the FC in frequency, unique CDR3 number, and mutation rate each lineage. For lineage frequency, the number of increased lineages with FC >3 was greater than that of decreased lineages at both 48hr and 72hr post-stimulation in both IRMs and AGMs (**Figures 3A, D**). For the unique CDR3 number per lineage, the number of increased lineages with FC >2 was significantly higher than that of decreased lineages at 48hr post-stimulation in both species (**Figures 3B, E**). Regarding mutation rate, the number of increased lineages exceeded that of decreased lineages at most timepoints including as early as 8hr post-stimulation in both species (**Figures 3C, F**), which may be attributed to the expansion of B cells originating from memory-like B cells with high-mutation rates. Additionally, in the IgH repertoire of IRMs, increased and decreased lineages exhibited similar results including the direct distribution and FC-based distributions across the three characteristics (**Supplementary Figure S14, S15**). Collectively, these results demonstrate that TLR7/8 stimulation induces the expansion of a subset of antibody lineages through increased frequency, clonotype count and mutation rates, particularly at 48hr and 72hr post-stimulation.

### Identifying TLR7/8 agonist-induced expanded lineages at 48hr and 72hr post-stimulation

**Figure 4:**
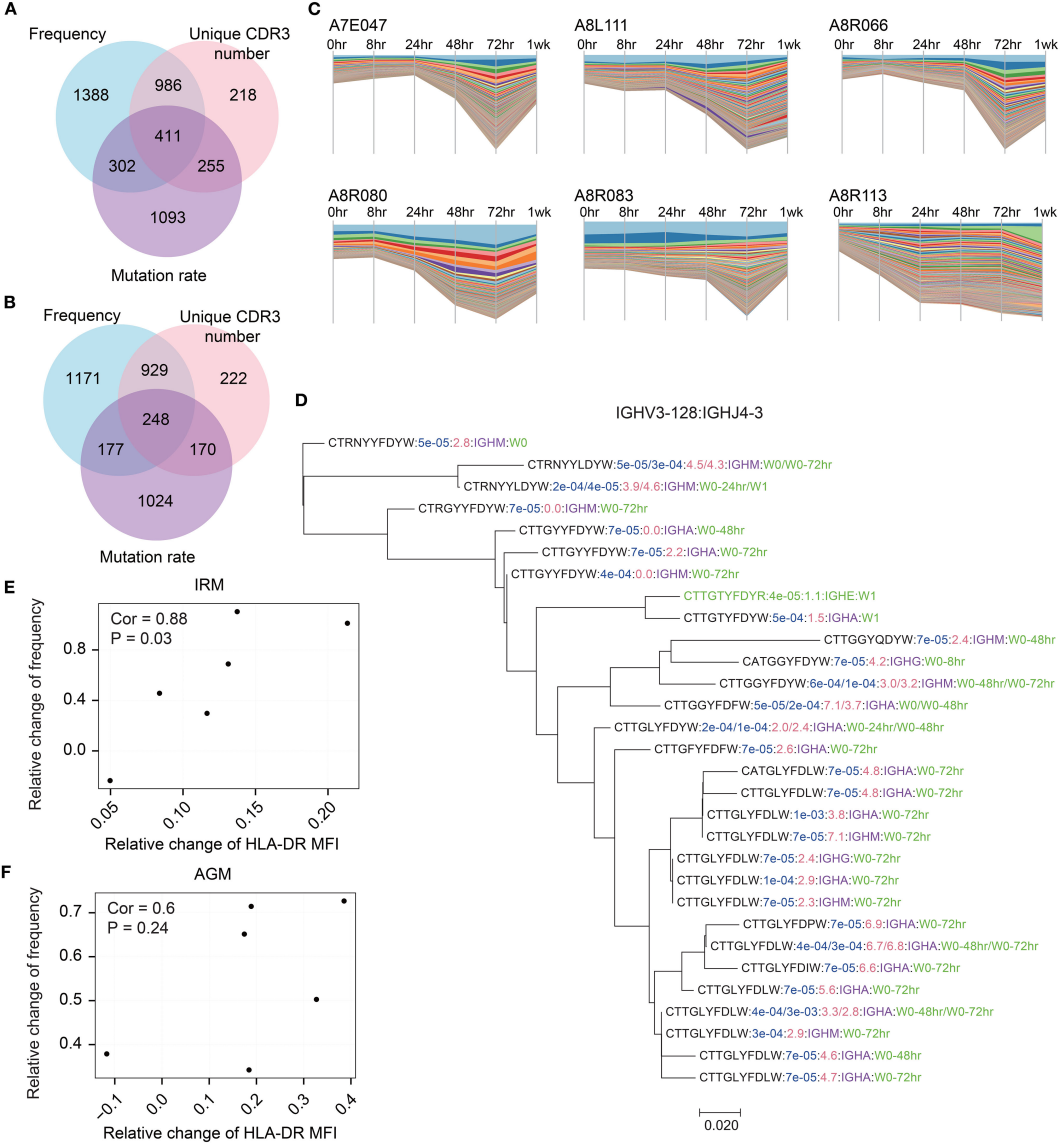
Identification of TLR7/8 agonist-induced expanded lineages and their association with B cell activation. **(A,B)** Overlap of IgKL expanded lineages that met the thresholds for frequency, unique CDR3 number, and mutation rate in IRMs**(A)** and AGMs **(B)**. **(C)** Dynamics changes in antibody frequency of IgKL chain expanded lineages across timepoints. Each bar represents a unique antibody, with bar width corresponding to antibody frequency. **(D)** The phylogenetic tree displayed an expanded IgH lineage. Sequences containing the CDR3 region, J gene region, and a 10-bp non-CDR3 segment of the V gene region were used for construction. All sequences in this lineage belonged to IGHV3–128 and IGHJ4-3. Blue text indicates sequence frequency in the sample; red text, sequence mutation rate; purple text, isotype; and green text, samples containing the sequence. **(E, F)** Spearman’s correlation between changes in the frequency of IgKL chain expanded lineages and changes in HLA-DR expression in IRMs **(E)** and AGMs **(F)**. relative change of frequency was calculated as (frequency at post-stimulation – frequency at pre-stimulation)/frequency at pre-stimulation.

Since we observed an increase in lineage distributions following TLR7/8 stimulation, we next aimed to identify the significantly expanded lineages at 48hr and 72hr timepoints as the TLR7/8 agonist-induced antibodies, based on the three predefined characteristics (see Methods). A total of 4653 expanded lineages were identified in IRMs, with 42.0% meeting the thresholds for two or three characteristics, including frequency, unique CDR3 number, and mutation rate (**Figure 4A**). Similarly, 3941 expanded lineages were identified in AGMs, with 38.7% meeting the thresholds for two or three characteristics (**Figure 4B**). Stream plots showed a consistent increase in expanded lineages over time, peaking at 72hr post-stimulation (**Figure 4C**; **Supplementary Figure S16**). To examine clonotype composition and dynamics, we constructed phylogenetic trees for several representative expanded lineages (**Figure 4D**; **Supplementary Figure S17**). The trees indicate that antibody clonotypes evolved from the 0hr sample to the 48hr and 72hr samples, underwent class switching from IgM to IgA, and accumulated somatic mutations, resulting in increased mutation rates (**Figure 4D**). To assess the identified expanded lineages, we examined the correlation between their frequencies and B cell activation, as reflected by the significant increase in HLA-DR expression in both IRMs and AGMs (**Figure 1B**). The changes of lineage frequencies were positively correlated with HLA-DR changes in IRMs (**Figure 4E**), while AGMs exhibited a similar trend of positive correlation (**Figure 4F**). The findings suggest that the TLR7/8 agonist-induced expanded lineages are associated with B cell activation.

### TLR7/8 agonist stimulation induces λ chain bias and divergent antibody responses across individuals

**Figure 5:**
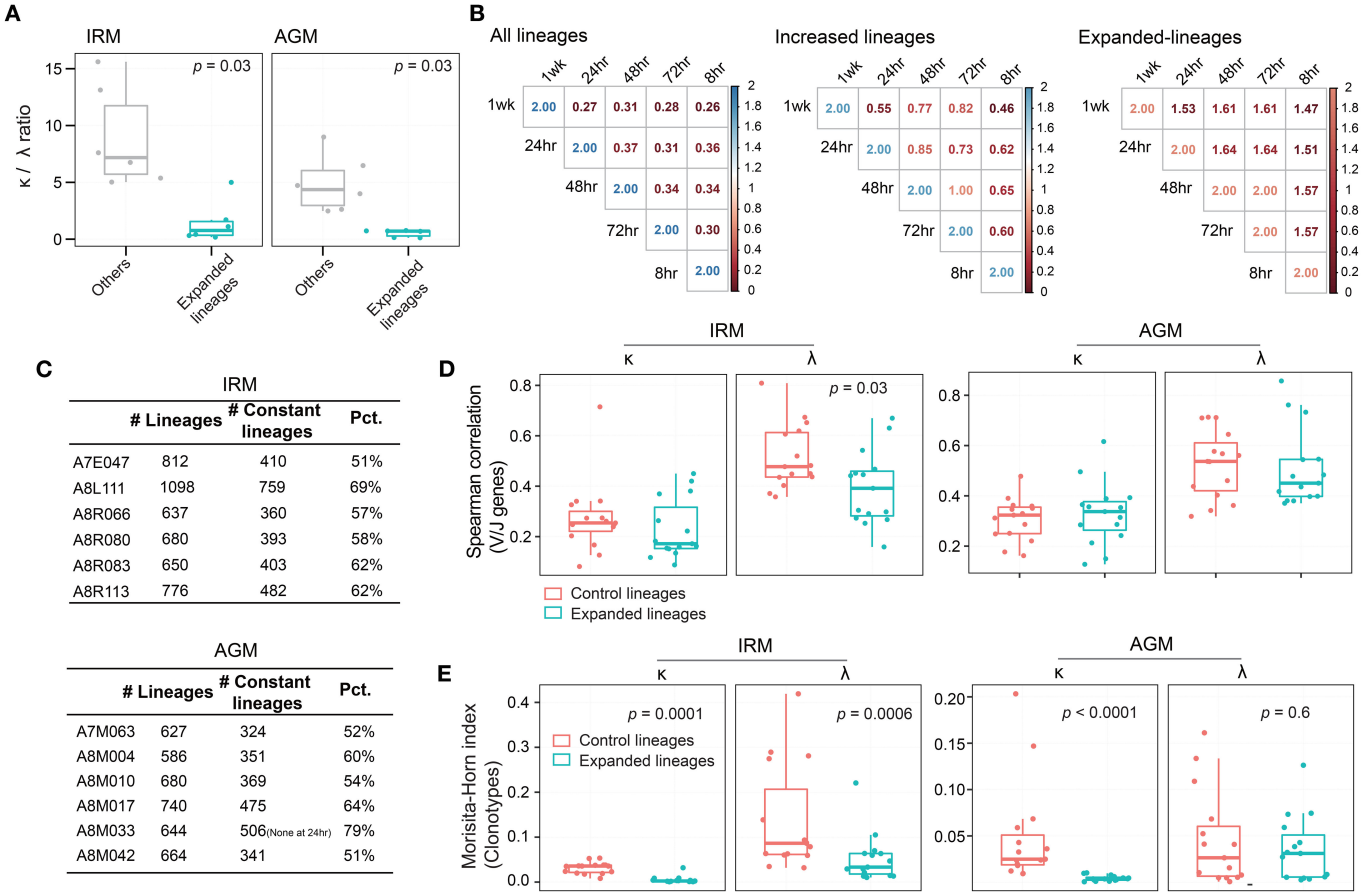
Characteristics of IgKL chain expanded lineages induced by TLR7/8 stimulation. **(A)** κ/λ ratio of expanded lineages and control lineages at 72hr. **(B)** Lineage overlap index between any two timepoints. **(C)** Number of expanded lineages in both IRMs and AGMs. “Constant lineages”, refers to lineages present across all timepoints. “Pct.”, represents the percentage of constant lineages among expanded lineages. **(D)** Inter-individual similarity in V/J gene usage evaluated using Spearman’s correlation for expanded and control lineages in both IRMs and AGMs. **(E)** Inter-individual similarity in antibody clonotypes evaluated using Morisita–Horn index for expanded and control lineages in both IRMs and AGMs. (Paired Wilcox-ranked test).

We analyzed the characteristics of the expanded lineages following TLR7/8 agonist stimulation. First, we examined the composition of light chain. The κ/λ ratio of expanded lineages was significantly lower than that of randomly sampled lineages with an identical frequency distribution in both IRMs and AGMs (**Figure 5A**), suggesting a preferential usage of λ chain in response to TLR7/8 stimulation. Second, we assessed the similarity of expanded lineages across timepoints. Overlap indices were calculated between each pair of timepoints for each individual, revealing that expanded lineages showed the highest overlap index compared to both the all lineage pool and increased lineages (**Figure 5B**; **Supplementary Figure S18**). Furthermore, more than half of expanded lineages (> 51%) were present at all timepoints (**Figure 5C**), indicating that these lineages were consistently maintained over one week. Third, we examined the similarity of expanded lineages across individuals. Since disease and vaccine stimulation could elicit convergent antibodies across individuals to target antigens (19, 27, 42), it is important to determine whether TLR7/8 agonists, as potential vaccine adjuvants, induce convergent or divergent antibody responses. To serve as a control, we selected a set of control lineages with the same κ/λ ratio and the same number of lineages as the expanded lineage group. We then compared the expanded lineages to the control lineages based on both gene usage similarity and clonotype similarity. For gene usage, correlation coefficients between individuals were not significantly different, and IRM λ-originated expanded lineages exhibited even lower correlation values (**Figure 5D**). Regarding clonotypes, the inter-individual similarity of expanded lineages was significantly lower than that of control lineages, except for AGM λ-originated expanded lineages (**Figure 5E**). These findings demonstrate that the expanded lineages induced by TLR7/8 stimulation were highly divergent antibodies, and so the TLR7/8 agonist could provide a broad and diverse immune foundation when used as an adjuvant.

### TLR7/8 agonist-induced expanded lineages primarily originate from memory-/effector-like B cells

**Figure 6:**
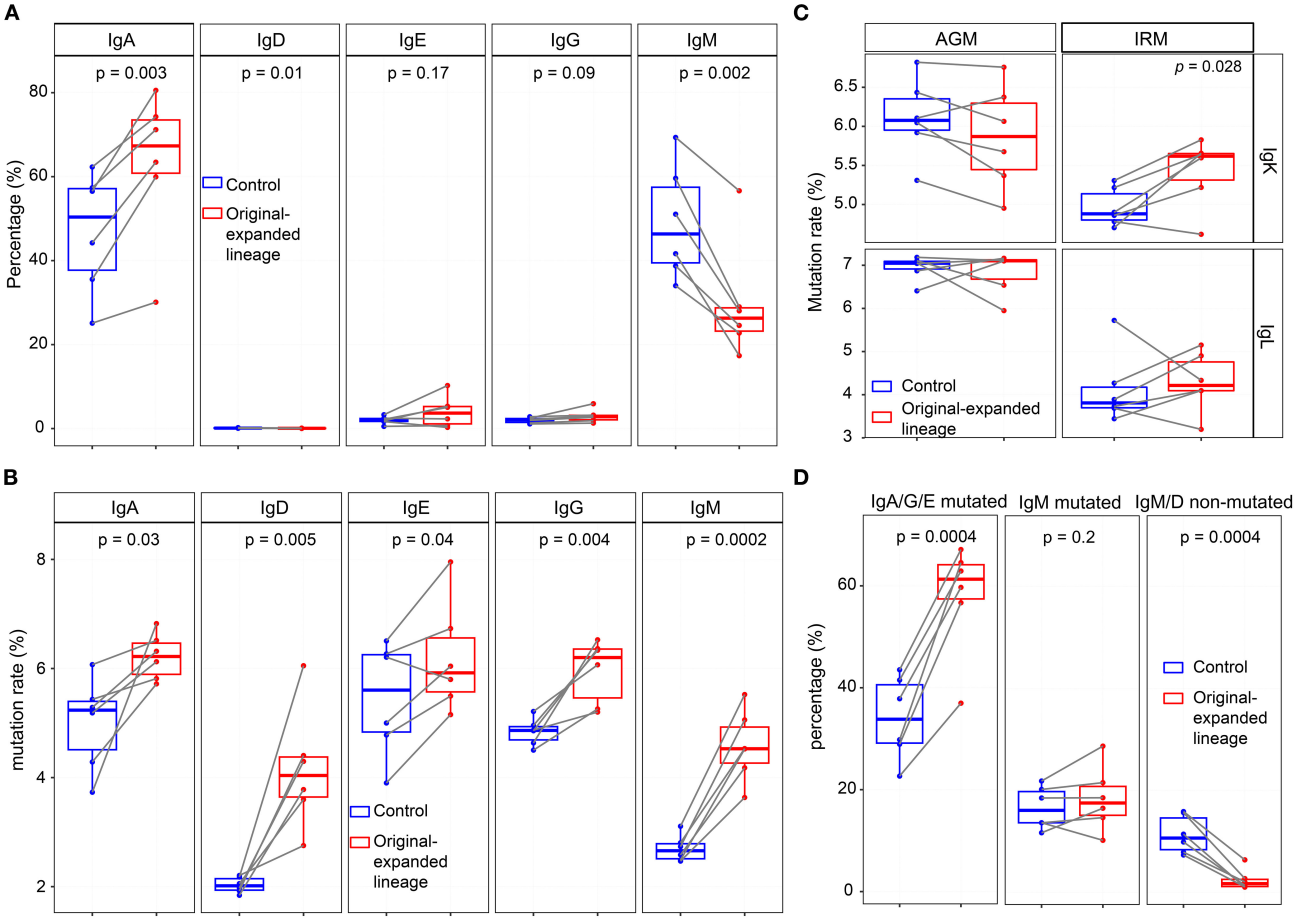
Mutation rate and isotype distribution of original-expanded lineages at 0hr. **(A)** Frequency of IgH isotypes in original-expanded and control lineages. **(B)** Mutation rates of each IgH isotype in original-expanded and control lineages. **(C)** Mutation rates of IgKL in original-expanded and control lineages. **(D)** Frequency of IgA/G/E mutated and IgM/D non-mutated sequences in the original-expanded and control lineages. IgA/G/E mutated, IgA, IgG, or IgE with a mutation rate > 3%. IgM mutated, IgM with a mutation rate >3%. IgM/D non-mutated, IgM or IgD with a mutation rate<0.5%. (Paired Wilcox-ranked test).

The proportion of IgG within expanded lineages decreased after stimulation, whereas the proportions of other isotypes remained stable across timepoints (**Supplementary Figure S19A**). In IgH expanded lineages, the proportion of IgM/IgD non-mutated sequences declined from 48hr to 1wk, while IgA/IgG/IgE mutated sequences showed a modest increase at 48hr (**Supplementary Figure S19B**). For IgKL expanded lineages, the proportion of non-mutated sequences decreased significantly after stimulation, whereas the proportion of mutated sequences increased significantly (**Supplementary Figure S19C**). Together, these results indicate that expanded lineages are characterized by elevated somatic mutation rates. To investigate the status of original B cells affected by TLR7/8 simulation, we tracked and analyzed the antibody sequences from the expanded lineages at the pre-stimulation (0hr), referred to as original-expanded lineages. The analysis included mutation rate and IgH isotypes, which provided insights into the status of the B cells. At 0hr, except for the original-expanded lineages, we randomly sampled the equal number of lineages from the remaining lineages as controls. Compared with the controls, the original-expanded lineages contained more IgA antibodies and fewer IgM antibodies (**Figure 6A**). Since antibodies from memory B cells have higher levels of SHM, while antibodies from naïve B cells have lower levels of SHM (43), we examined the mutation rates in different isotype of heavy chain and light chain. As a result, all isotype antibodies in original-expanded lineages exhibited significantly higher mutation rates, including IgM (**Figure 6B**). Interestingly, although IgM antibodies are generally from naïve B cells with very low mutated rates, the high-mutated IgM antibodies in these lineages may originate from IgM memory B cells (44). Within the IgK repertoire in IRM, mutation rates were higher in originally expanded lineages than in controls (**Figure 6C**). Antibodies were grouped into three origin categories—IgA/G/E-mutated, IgM-mutated, and IgM/G non-mutated—as proxies for memory/effector-like, IgM memory-like, and naïve B cells (45). IgA/G/E-mutated sequences dominated the expanded lineages (60%), significantly more than in controls; ~20% were mutated IgM (**Figure 6D**). In summary, our data suggest that TLR7/8 stimulation primarily activates memory- and effector-like B cells in peripheral blood; however, functional validation will be required in future studies.

## Discussion

Previous studies have primarily focused on the cellular responses to TLR7/8 agonist stimulation, often overlooking the broader impact on the response of antibody repertoire in B cells. Since TLR7/8 agonists are potential vaccine adjuvants, research on antibody repertoire dynamics can offer valuable insights for adjuvant selection during vaccine development. Our study demonstrates that administration of a TLR7/8 agonist alone skews the antibody repertoire in NHPs within one week. Following TLR7/8 stimulation, immune cells in peripheral blood were redistributed, and B cells were activated and proliferated at 24hr. However, changes of V/J genes, clonotypes, and lineages were not clearly observed until 48hr post-stimulation. In IRMs, an increase in both repertoire diversity and the number of increased lineages was observed between 48hr and 1wk, while in AGMs, this increase occurred between 48hr and 72hr, with a recovery observed at 1wk. The expanded lineages induced by TLR7/8 agonists were identified, and the antibodies in these expanded lineages exhibited a λ chain bias, and were broadly divergent. These lineages were predominantly derived from highly mutated IgA/G/E and highly mutated IgM antibodies, suggesting an origin from memory- and effector-like B cells; however, functional validation is still required. This broad B cell profile contributes to a comprehensive adaptive immune response, supporting antigen stimulation in vaccine contexts. TLR7/8 agonist stimulation rapidly activates B cells within 48hr, reaching peak proliferation at 72hr, aligning with the timing of the largest antibody responses observed following vaccination, which typically occur on days 7 and 8 post-vaccination (27, 45). The expansion of divergent B cells increases the likelihood that antigens will quickly and accurately recognize and bind the appropriate B cells to induce a robust vaccine response.

Due to the process of V(D)J rearrangement and antibody affinity maturation, the antibody repertoire exhibits vast diversity. We have developed an antibody repertoire sequencing method for monkeys, capable of generating millions of antibody sequences for each sample (21–23). Using this approach, we could systematically monitor the dynamic changes in the antibody repertoire before and after TLR7/8 agonist stimulation. Although each sample generated millions of sequences and more than ten thousand unique antibodies, identifying the subset of antibodies associated with TLR7/8 stimulation remains challenging. For peptides or antigens, their associated antibodies can be accurately identified by sorting B cells that directly bind to the peptides or antigens (27, 46). However, unlike antigens, TLR7/8 agonists theoretically do not induce a specific antibody response but rather elicit a broad and diverse antibodies repertoire, which also have been demonstrated by our data. Thus, we employed bioinformatic method to infer the associated antibodies. We first defined lineages by clustering similar clonotypes originating from the same B cell source, as previously applied it in other studies (21, 27, 28, 47). Using pre-stimulation data as a baseline, we then analyzed the distributions of increased and decreased lineages to find out distinguishing characteristics such as frequency, antibody number, and mutation rate. The TLR7/8 agonist-induced expanded lineages were identified by the characteristics. Since the monkeys underwent no other interventions, the shared characteristics could be attributed to TLR7/8 stimulation, supporting the validity of this method for identifying expanded lineages. The method can also be used to vaccine evaluation studies or immune related disease research using longitudinal immune repertoire sequencing data.

We deliberately included both IRM and AGM to broaden the translational relevance of our findings. IRM is a standard NHP model for vaccine research, whereas AGM is a less common but complementary model. Most wild AGMs control simian immunodeficiency virus (SIV) replication without progression to disease, and AGMs have therefore been extensively used to study immunodeficiency, including HIV immunity and vaccine responses (21, 48–50). Because many HIV vaccine programs employ African green monkeys to evaluate immunogenicity and adjuvant activity, our observations of TLR7/8 agonist effects on B cells may help determine whether TLR7/8 agonists are suitable adjuvant candidates in AGM-based HIV vaccine studies and, by extension, inform expectations for other NHP models. Beyond species-specific insight, demonstrating concordant repertoire and lineage dynamics across two distinct NHP species increases confidence that the reported B-cell responses reflect biologically robust effects of TLR7/8 stimulation rather than idiosyncrasies of a single model. In our study, although IRMs and AGMs exhibited similar immune responses to TLR7/8 stimulation, some differences were also observed. For example, the proportion of KI67+ B cells increased at 24hr in IRMs but showed no significant change in AGMs; antibody repertoire diversity remained elevated at 1wk in IRMs but returned to pre-stimulation levels in AGMs. AGMs, as natural hosts of simian immunodeficiency virus, have been reported to exhibit relatively weak TLR7 signaling in DCs and low IFN-I secretion in response to ssRNA and TLR7/8 agonists (49–52). This difference in innate immune responses to TLR7 stimulation may influence their adaptive immune responses (53). Thus, the differing responses of IRMs and AGMs to the TLR7/8 agonist are likely associated with differences in IFN-I production; however, further studies are required to establish a direct link.

In this study, comparisons between IRMs and AGMs were focused on IgKL repertoire, as IgH repertoire sequencing data were not available for AGMs. Compared to the IgKL repertoire, IgH repertoire, which contain D genes, exhibits greater sequence diversity and includes information on antibody isotypes. However, the public database, including the IMGT database, lack most germline V and C gene sequences, making it challenging to design PCR primers for capture. Attempts to use homologous IRM primers for AGM IgH repertoire sequencing resulted in a high degree of non-specific amplification, and the data quality did not meet the criteria for further analysis (data not shown). Additional efforts are needed to identify the germline V, D, J, and C gene sequences. We previously developed the IMPre software to infer germline genes (26) and identified novel V/J antibody genes in IRM (22). In the future, with a more complete germline gene database, IgH repertoire sequencing in AGMs could be employed for comparative studies with IRMs and other primate models.

A limitation of our study is the absence of a co-administered protein antigen, which prevents direct assessment of how TLR7/8 stimulation interacts with antigen-specific BCR signals to shape clonal selection and differentiation. While TLR signaling broadly activates B cells, antigen-specific B cells receiving both BCR and TLR signals preferentially expand and survive. Thus, our findings primarily reflect the effects of TLR7/8 stimulation alone. Vaccine adjuvant applications, such as an interested antigen with the TLR7/8 agonist, require systematic evaluation in antigen-containing formulations. Furthermore, our conclusions are primarily based on antibody repertoire sequencing and therefore require additional experimental validation at both the protein and functional levels. Complementary approaches could directly test and extend our findings. For example, flow cytometry with anti-kappa and anti-lambda antibodies could confirm any λ-chain bias at the protein level; quantitative measurement of isotype-specific antibody titers by ELISA and related serological assays could determine whether TLR7/8 agonists induce isotype bias; and conventional flow-cytometric panels incorporating memory and effector markers could be used to validate changes in B-cell subset composition before and after stimulation, thereby confirming the origin of TLR7/8 agonist–elicited cells. In addition, single-cell multimodal profiling—for instance, single-cell RNA-seq paired with V(D)J sequencing—would allow simultaneous interrogation of clonal identity and cell state, distinguishing broad repertoire diversification from expansion of a limited set of antibodies. Although these experiments were beyond the scope of the present study and constrained by sample availability from the original cohorts, they represent feasible and important follow-up investigations that would substantially strengthen the mechanistic interpretation and translational relevance of our findings.

## Data Availability

The datasets presented in this study can be found in online repositories. The names of the repository/repositories and accession number(s) can be found below: https://db.cngb.org/data_resources/project, CNP0000866.
