# Supplementary material for: Toll-like receptor 7/8 agonist stimulation rapidly skews the antibody repertoire of B cells in non-human primates
Source: Front Immunol. 2025 Sep 22;16:1647209. doi: 10.3389/fimmu.2025.1647209 (PMC12497728; doi:10.3389/fimmu.2025.1647209)
Supplement: Supplementary file 1 [file DataSheet1.docx]

**Table S1. NHP characteristics**

| **Group** | **Animal** | **Weight (kg)** | **Sex** | **Injection** |
| --- | --- | --- | --- | --- |
| RM | A8R080 | 4.80 | F | IDR-053 (2.5mg) |
|  | A8R113 | 5.40 | F |  |
|  | A8L111 | 6.75 | M |  |
|  | A8R083 | 6.40 | F |  |
|  | A8R066 | 5.80 | F |  |
|  | A7E047 | 4.85 | F |  |
|  | **avg wt.** | **5.67** |  |  |
| AGM | A8M004 | 5.65 | M | IDR-053 (2.5mg) |
|  | A8M017 | 5.15 | F |  |
|  | A8M033 | 5.15 | F |  |
|  | A8M042 | 4.70 | F |  |
|  | A7M063 | 4.50 | F |  |
|  | A8M010 | 6.30 | M |  |
|  | **avg wt.** | **5.24** |  |  |

These non-human primates were between four and five years old.

**Table S2.** **Antibodies for flow cytometry.**

| Antigen | Clone | Fluorophore | Supplier |
| --- | --- | --- | --- |
| CD3 | 10D12 | Biotin/SA PercPcy5.5 | Miltenyi primary/BD secondary |
| CD4 | L200 | PE-CF594 | BD |
| CD8 | SK1 | PE-Cy7 | BD |
| CD20 | LT20 | VioBlue | Miltenyi |
| HLA-DR | L243 | APC-H7 | BD |
| CD69 | FN50 | FITC | BD |
| ki67 | B56 | PE | BD |

**Table S3. Light chain primers.**

| Primer | Isotype | Group |
| --- | --- | --- |
| 5’-Bio ACACTTAATTAACATGGCGGGAAGATGAAGACA-3’ | κ | AGM & IRM |
| 5’-Bio ACACTTAATTAACAACGGAGTGACCGAGGGA-3’ | λ | AGM |
| 5’-Bio ACACTTAATTAACAACAGAGTGACTGACGGG-3’ | λ | IRM |
| 5’-Bio ACACTTAATTAACAACAGAGTGACCAAGGGG-3’ | λ | IRM |
| 5’-Bio ACACTTAATTAACAAVAGAGTGACCGWGGGG-3’ | λ | IRM |

**Table S4. Summary statistics of processed data (see accompanying Excel file).**

.


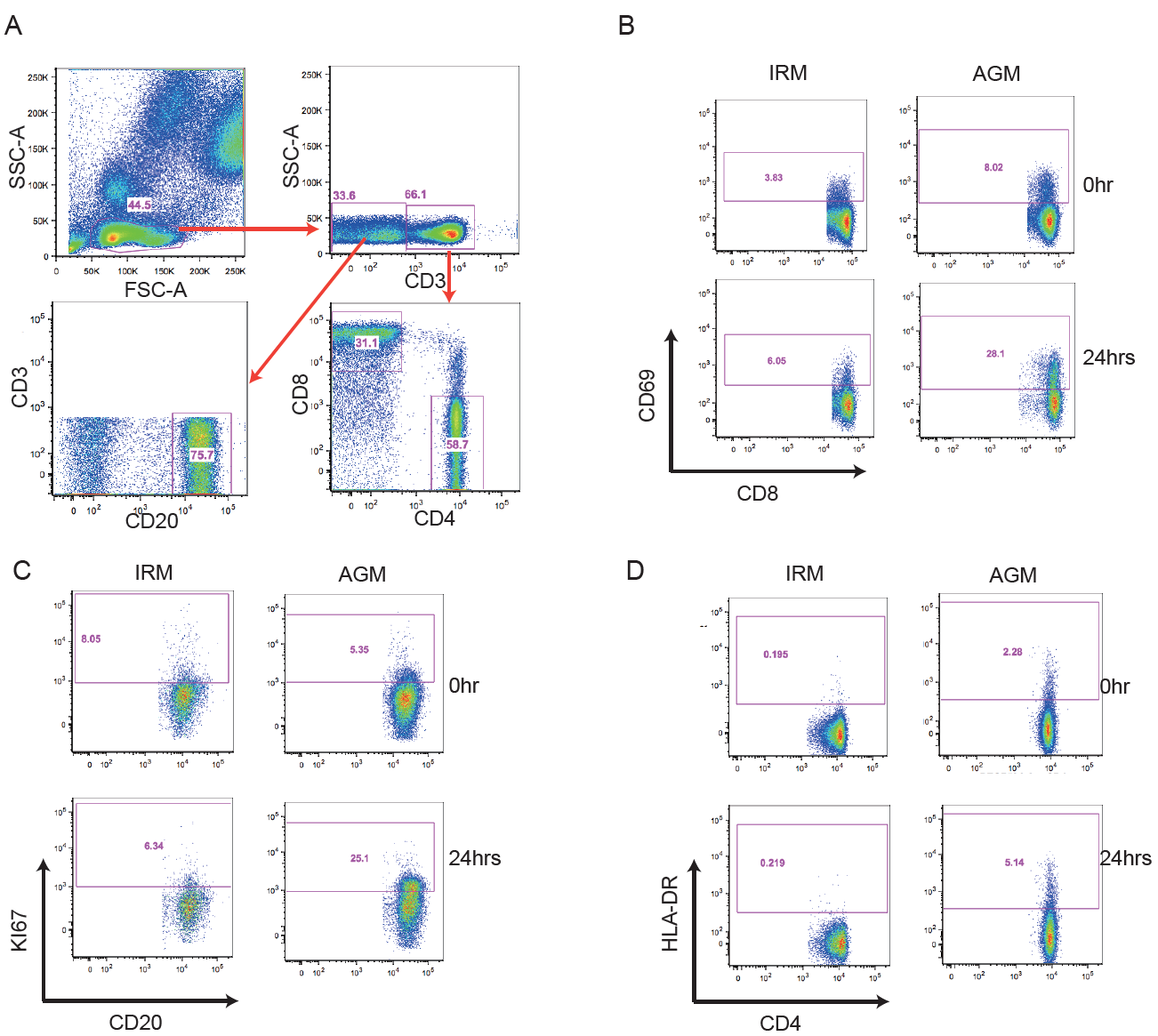


**Figure S1. Gating strategies of flow cytometry. (A)** A strategy for gating B-cell, CD4^+^ T cells, and CD8^+^ T cells. **(B)** A strategy for gating CD69^+^CD8^+^ T cells of IRMs and AGMs. **(C)** A strategy for gating Ki67^+^B cells of IRMs and AGMs. **(D)** A strategy for gating HLA-DR^+^CD4^+^ T cells of IRMs and AGMs.


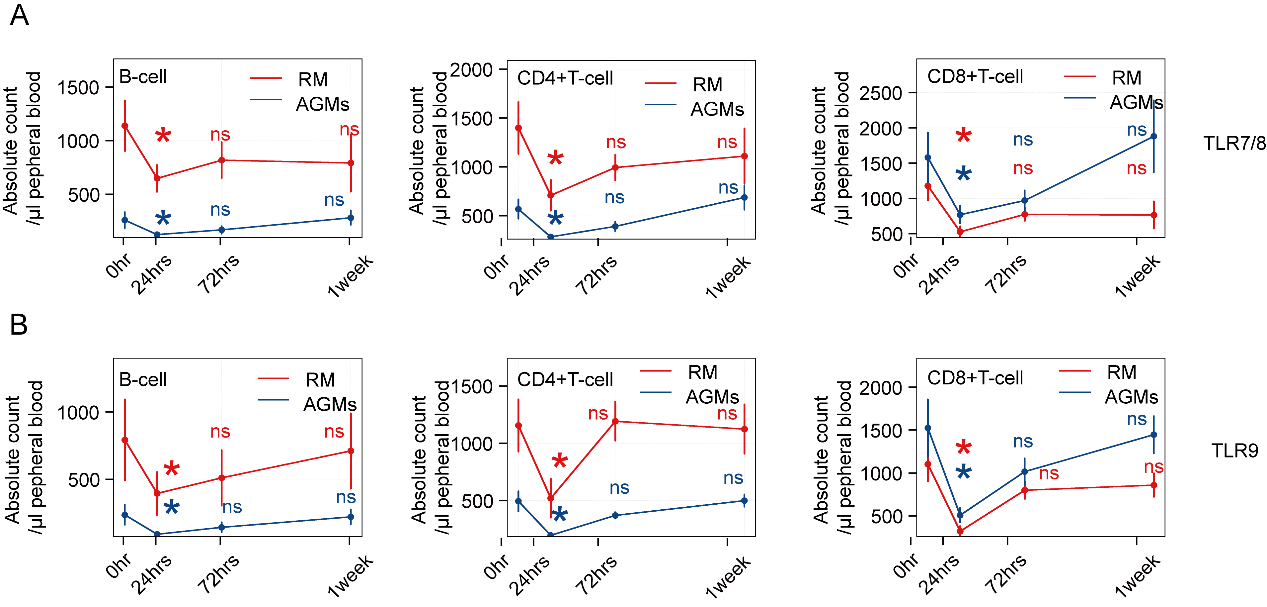


**Figure S2. Absolute count of B-cells, CD4^+^ T-cells, and CD8^+^ T cells.** (Paired Wilcox-ranked test, vs. 0hr, **p* < 0.05, *ns* *p* > 0.05).


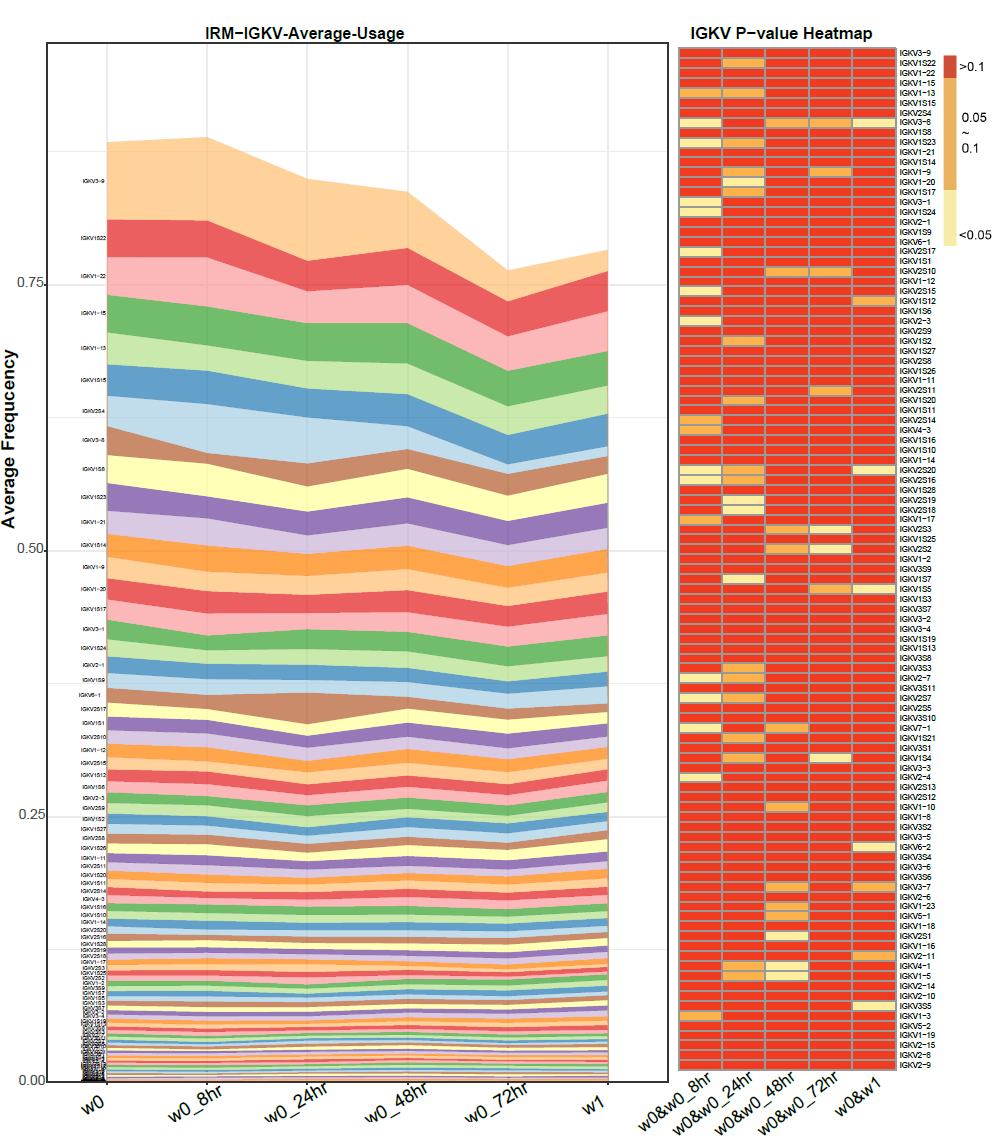


**Figure S3. Mean frequencies of IgK V-gene usage from IRM across all timepoints.** Heatmaps display adjusted p-values for comparisons of V-gene usage between W0 and each subsequent timepoint.


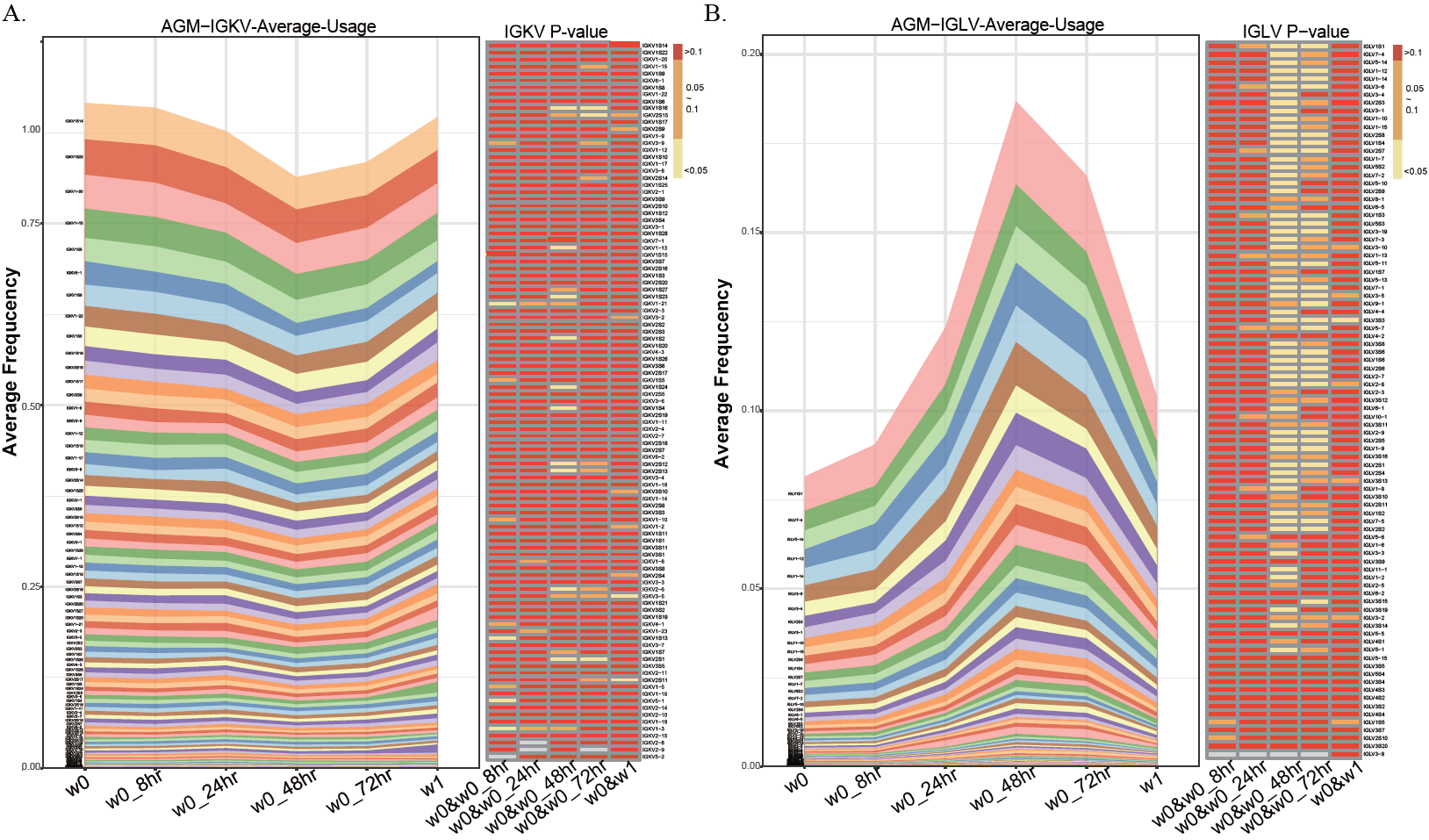


**Figure S4. Mean frequencies of IgK (A) and IgL (B) V-gene usage from AGM across all timepoints.** Heatmaps display adjusted p-values for comparisons of V-gene usage between W0 and each subsequent timepoint.


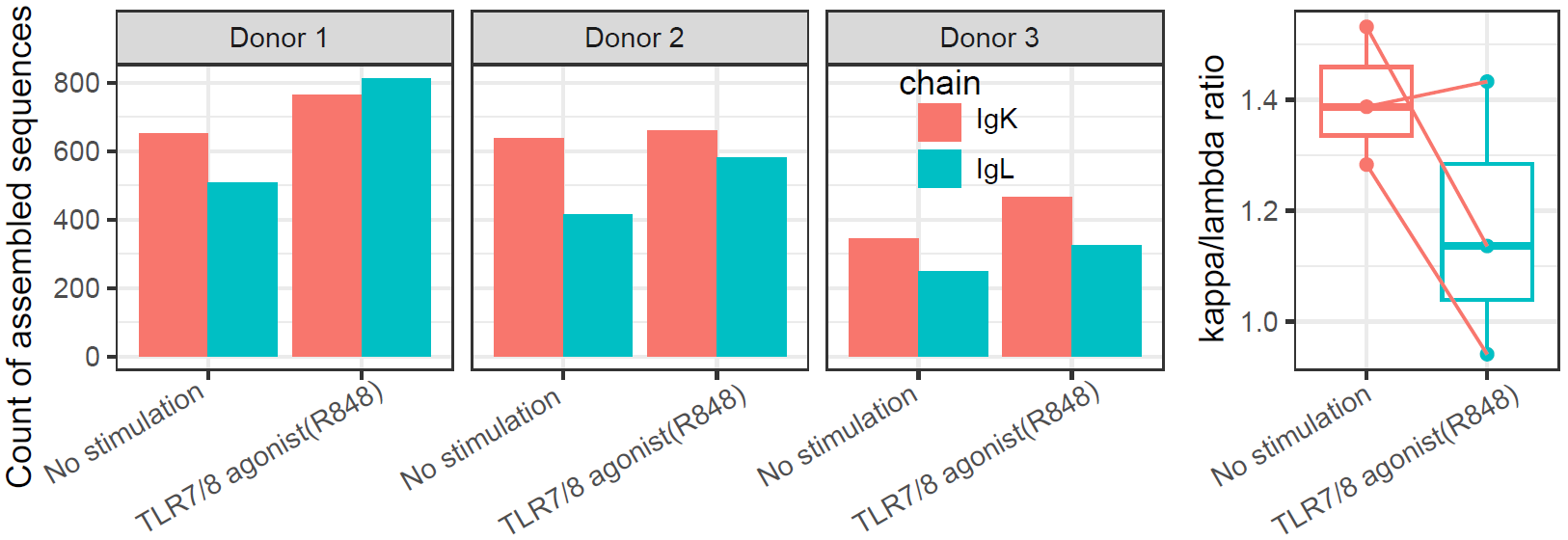


**Figure S5. Statistics of assembled IgK and IgL sequences from the RNA-seq data in Wu et al. study.** In that study, purified B cells from the PBMCs of three healthy donors were cultured either unstimulated or stimulated with a TLR7/8 agonist (R848), and RNA-seq was performed 24 hours after stimulation. Raw reads were assembled with TRUST4 to identify IgK and IgL sequences. Assembled sequences that contained a CDR3 and a V or J gene assignment were used to quantify IgK and IgL content and to calculate the kappa:lambda ratio.


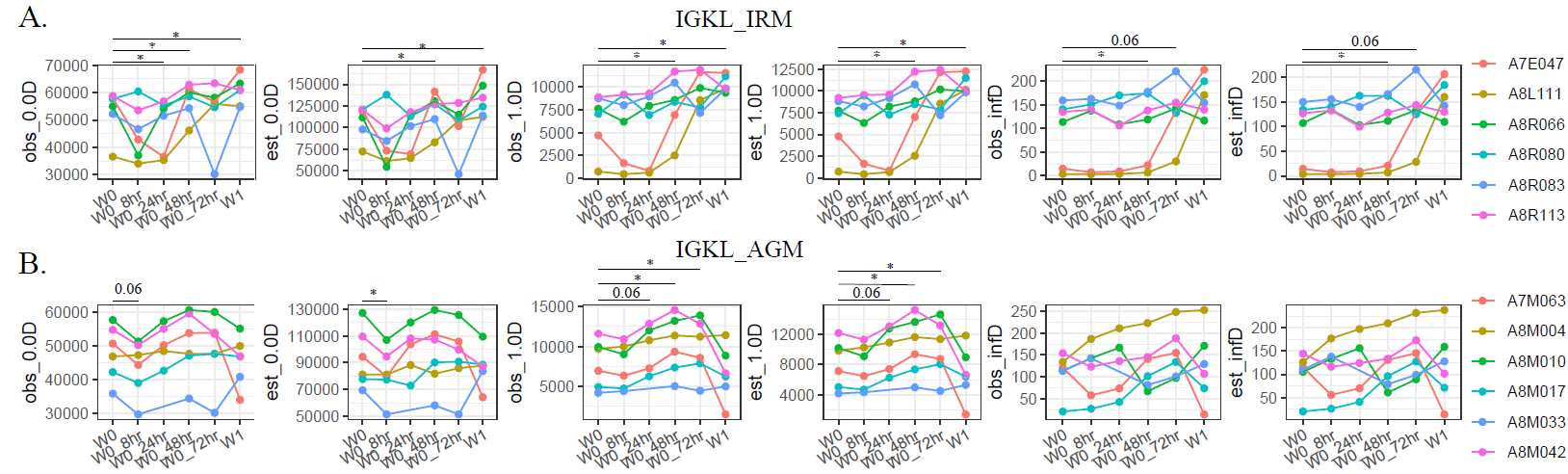


**Figure S6. Diversity metrics generated using the tool Recon.** (A) Diversity of IgKL in IRM. (B) Diversity of IgKL in AGM. Obs_0.0D, observed value with parameter Q = 0; est_0.0D, estimated value with parameter Q = 0; Obs_1.0D, observed value with parameter Q = 1; est_1.0D, estimated value with parameter Q = 1; Obs_infD, observed value with parameter Q = ∞; est_infD, estimated value with parameter Q = ∞. (*, p < 0.05).


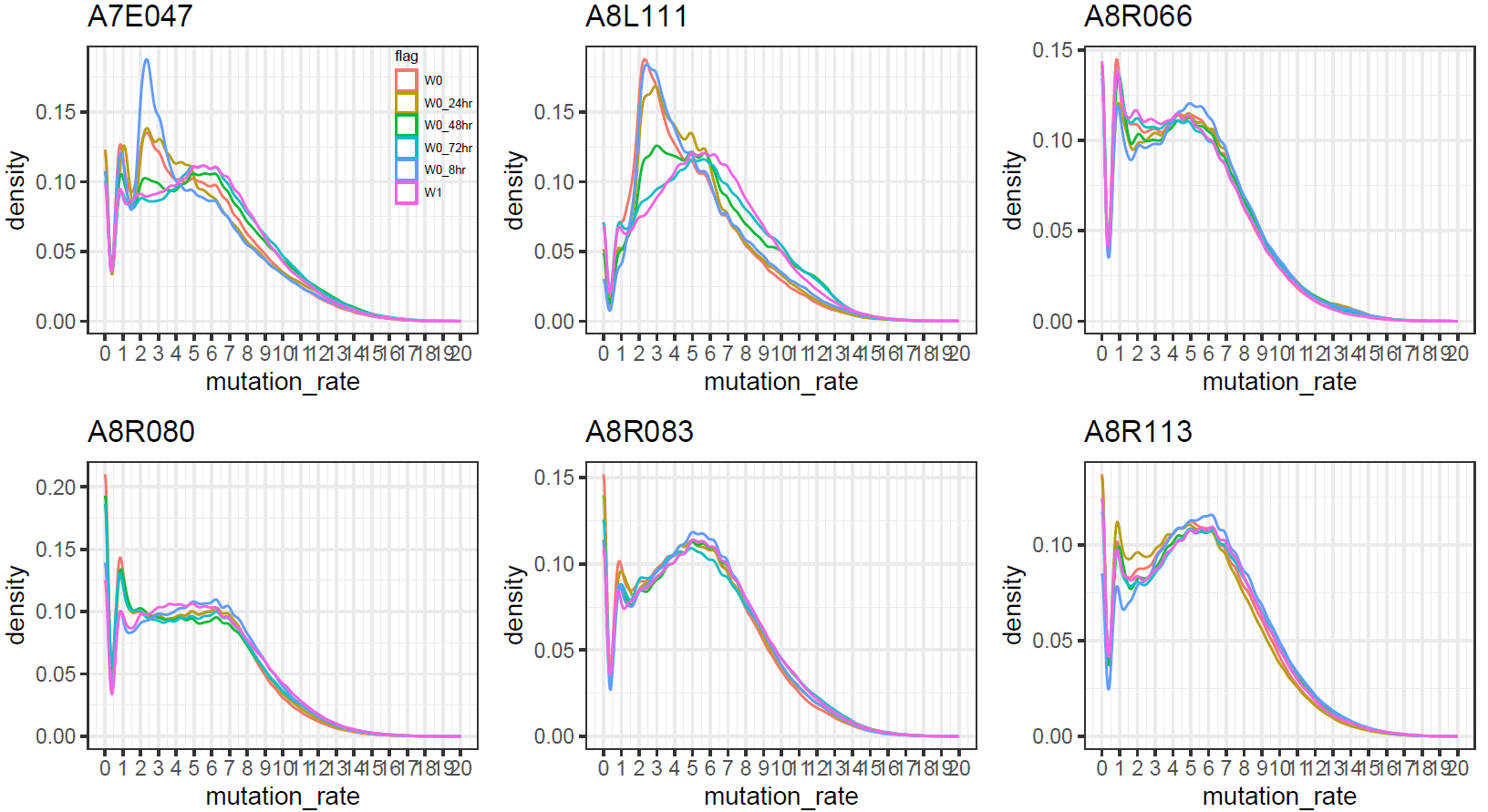


**Figure S7. Distribution of mutation rates in the IgKL repertoire across samples.**


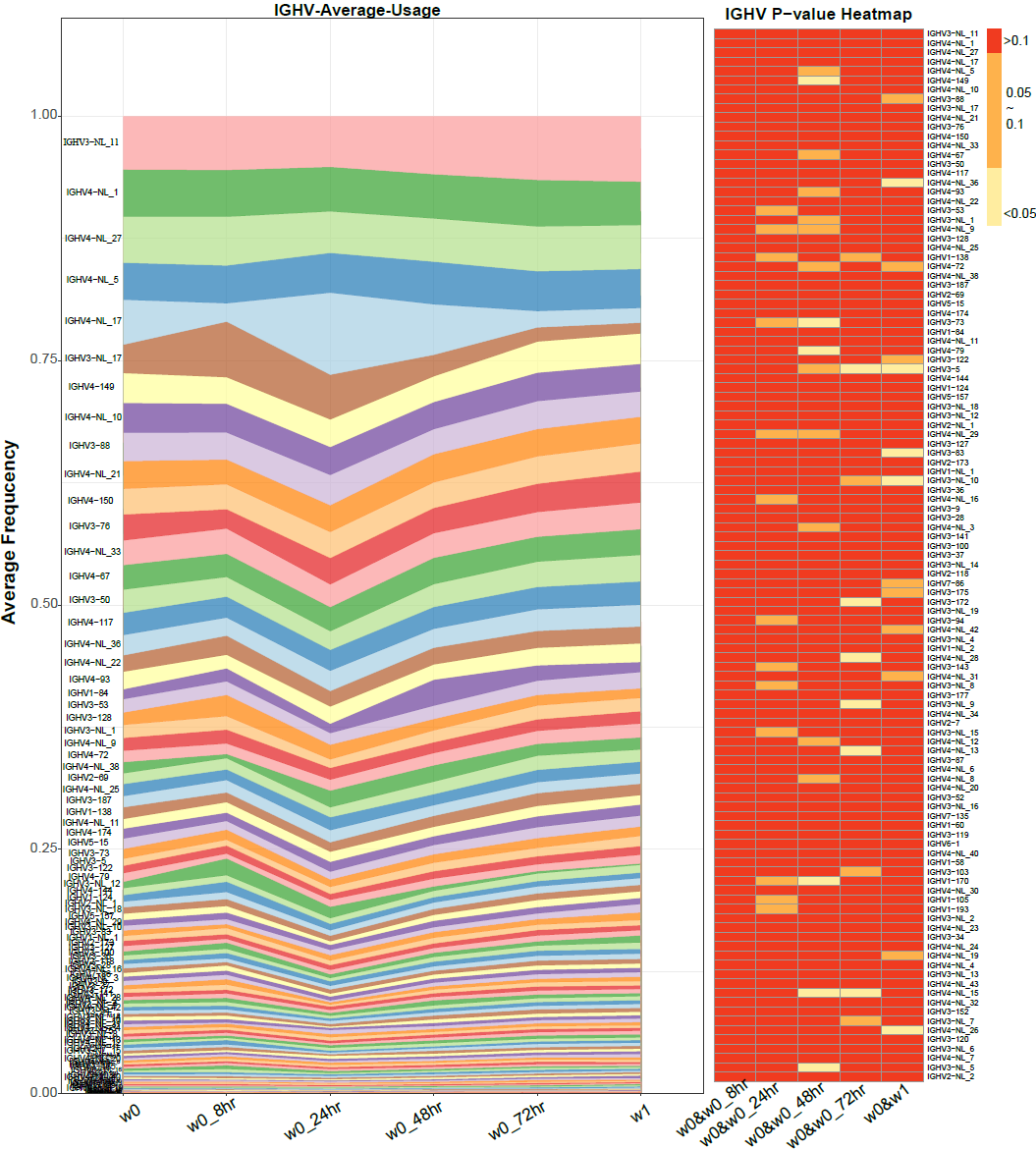


**Figure S8. Mean frequencies of IgH V-gene usage from IRM across all timepoints.** Heatmaps display adjusted p-values for comparisons of V-gene usage between W0 and each subsequent timepoint.


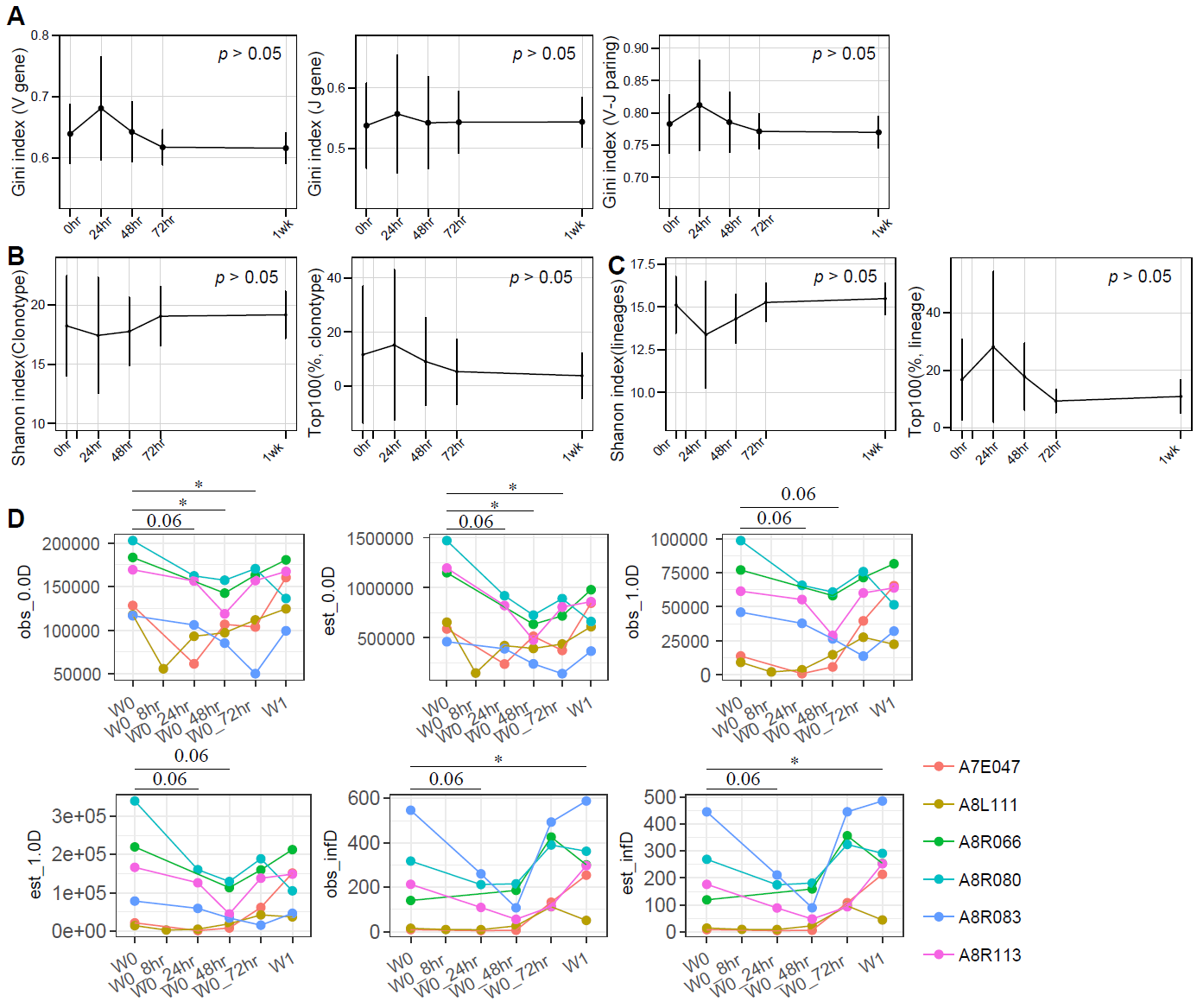


**Figure S9.** **Responses of IgH reperotiore to the TLR7/8 stimulation via TLR7/8 in IRMs. (A)** Gini-index of V- (left), J- (middle), and V-J paring (right) in IRMs at 0, 24, 48, and 72hr and 1wk post-stimulation via TLR7/8. **(B)** Shannon index (left) and Top100 of clonotypes (right) for IRMs at 0, 24, 48, and 72hr and 1wk post-stimulation. **(C)** Shannon index (left) and Top100 (right) of lineages for each IRM before and after stimulation. (D) Diversity metrics generated using the tool Recon. Obs_0.0D, observed value with parameter Q = 0; est_0.0D, estimated value with parameter Q = 0; Obs_1.0D, observed value with parameter Q = 1; est_1.0D, estimated value with parameter Q = 1; Obs_infD, observed value with parameter Q = ∞; est_infD, estimated value with parameter Q = ∞... (Paired Wilcox-ranked test. *, p < 0.05).


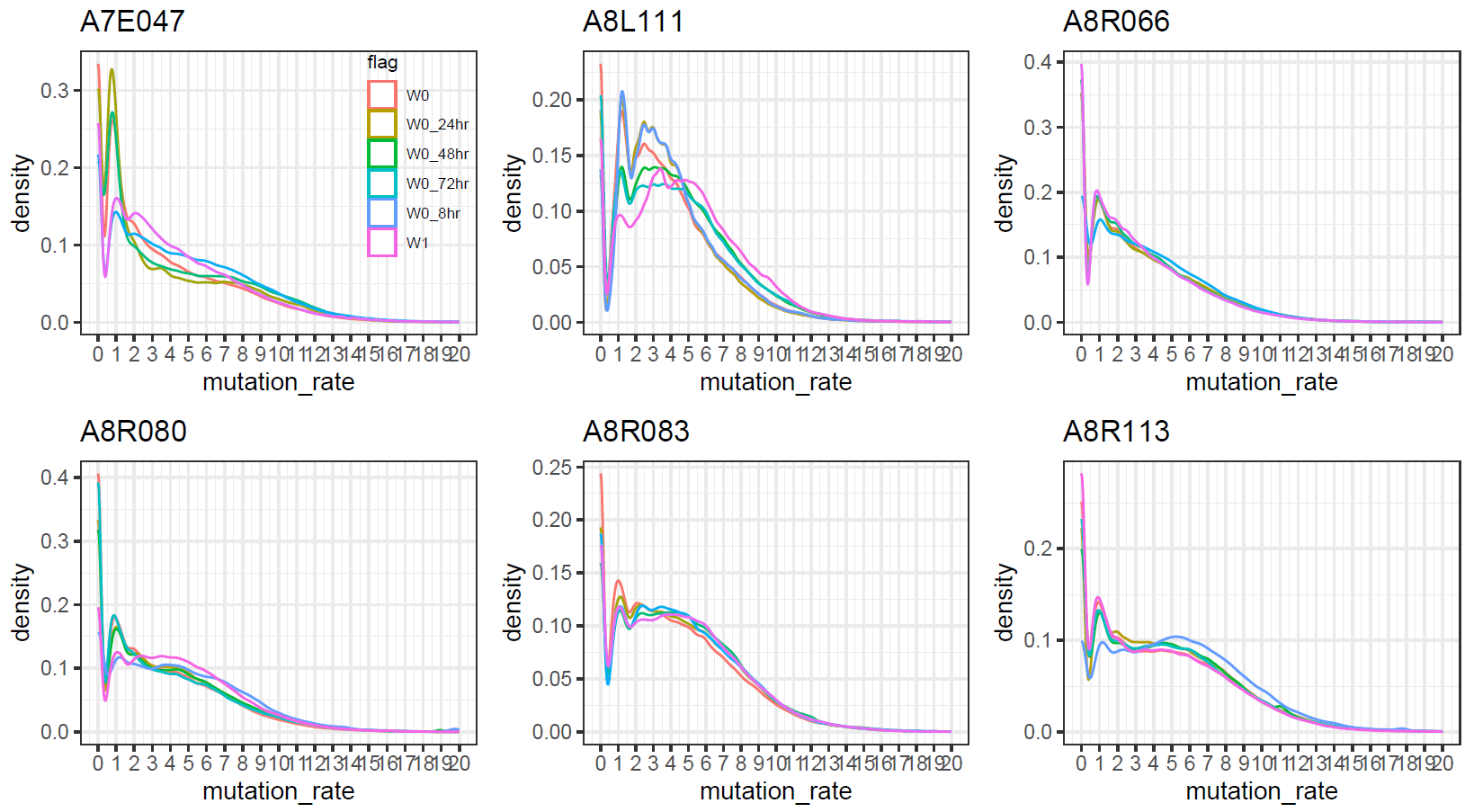


**Figure S10. Distribution of mutation rates in the IgH repertoire across samples.**
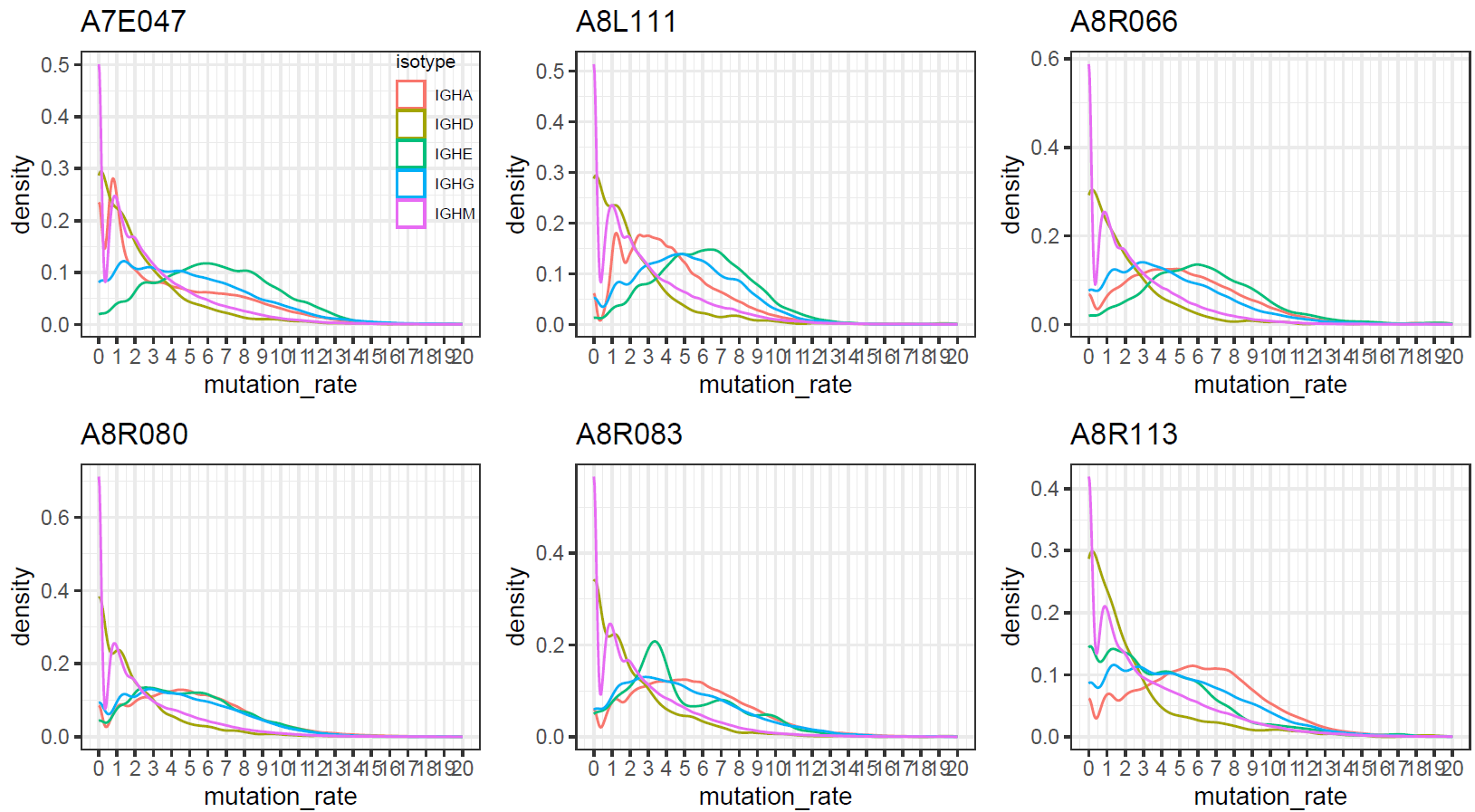


**Figure S11. Distribution of mutation rates in the IgH repertoire across isotypes. F**or each individual, only W0 sample was used.


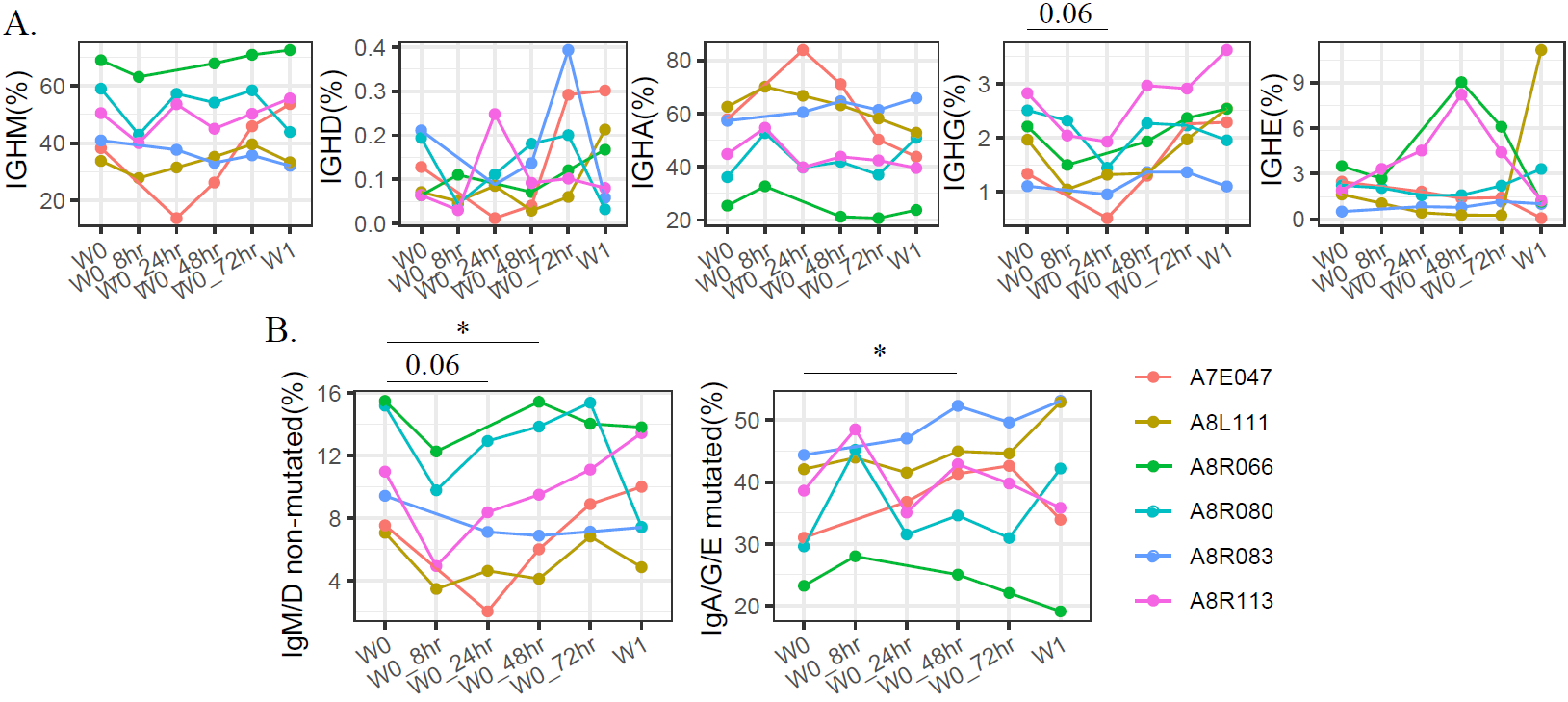


**Figure S12. Proportion of isotypes and mutation distribution**. (A) Proportion of each isotype in the IgH repertoire across all timepoints. (B) Proportion of IgM/D non-mutated sequences (IgM or IgD with mutation rates < 0.5%) and IgA/G/E mutated sequences (IgA, IgG, or IgE with mutation rates > 3%).
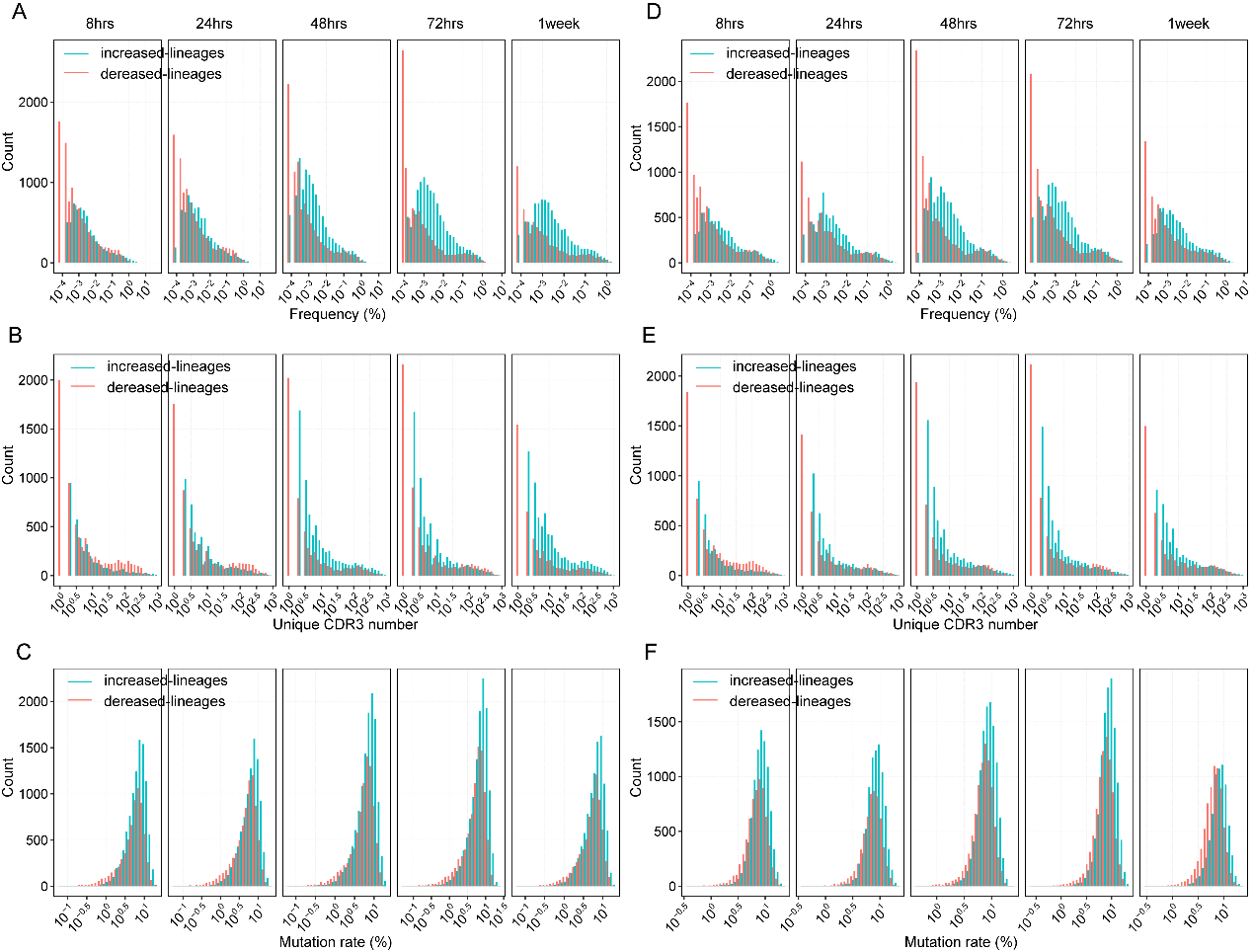


**Figure S13. Frequency, unique CDR3 number, and mutation rates of IgKL increased-lineages and decreased-lineages from 8hr to 1wk in IRMs and AGMs after the TLR7/8 stimulation**. Frequency of increased-lineages (blue) and decreased-lineages (red) at 8, 24, 48, and 72hr, and 1wk in IRMs **(A)** and AGMs **(D)**. Unique CDR3 number of increased-lineages (blue) and decreased-lineages (red) at 8, 24, 48, and 72hr, and 1wk post-stimulation in IRMs **(B)** and AGMs **(E)**. Mutation rate of increased-lineages (blue) and decreased-lineages (red) at 8, 24, 48, 72hr and 1wk post-stimulation in IRMs **(C)** and AGMs **(F)**.


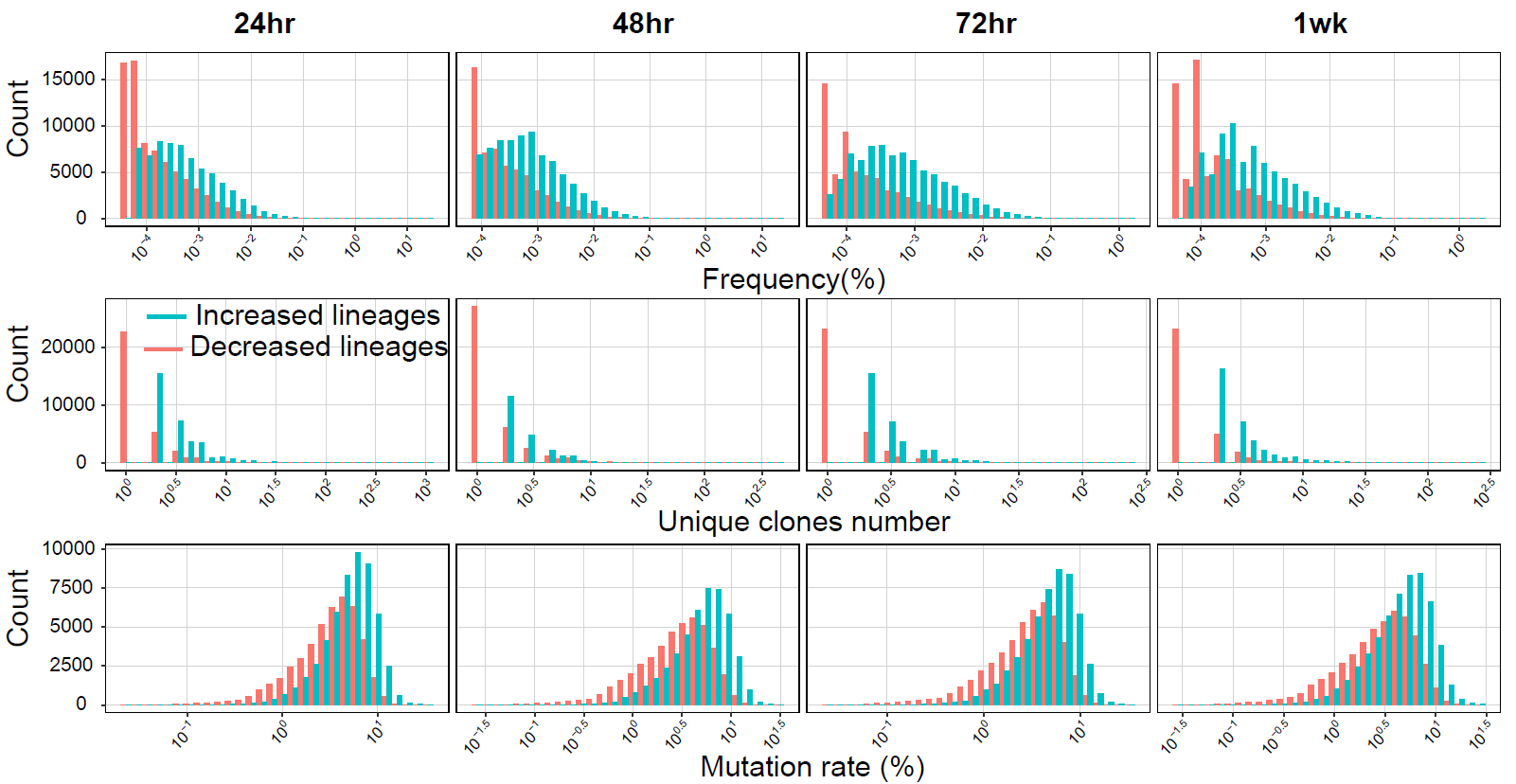


**Figure S14. Distribution of frequency, unique CDR3 and mutation rate of IgH increased and decreased-lineages of IRMs.** **(A)** Frequency of increased-lineages (blue) and decreased-lineages (red) at 8, 24, 48, and 72hr, and 1wk in IRMs. **(B)** Unique CDR3 number of increased-lineages (blue) and decreased-lineages (red) at 8, 24, 48, and 72hr, and 1wk post-stimulation in IRMs. **(C)** Mutation rate of increased-lineages (blue) and decreased-lineages (red) at 8, 24, 48, and 72hr, and 1wk post-stimulation in IRMs.


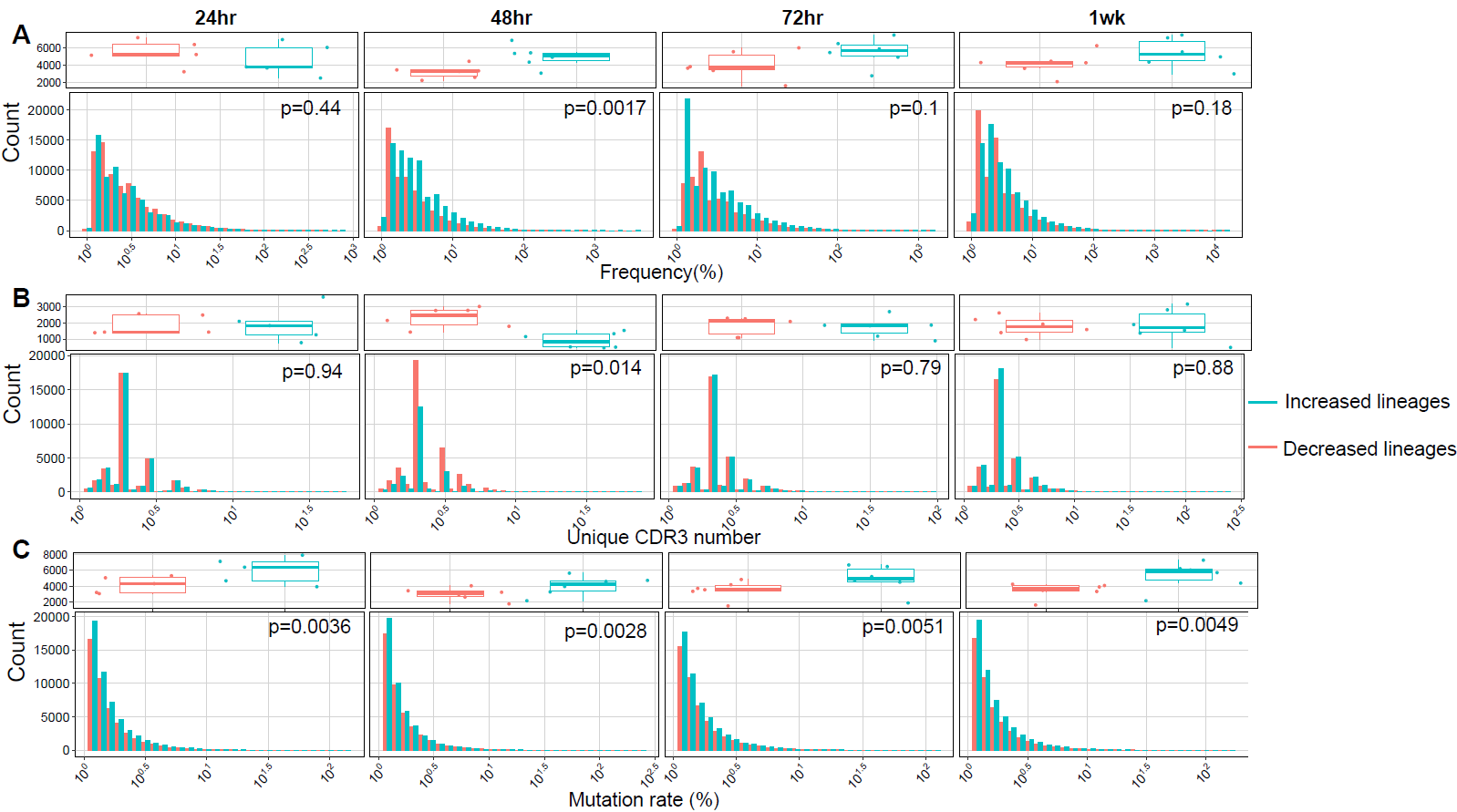


**Figure S15. FC distribution of frequency, unique CDR3 and mutation rate of IgH increased-lineages and decreased-lineages in IRMs.** **(A)** Frequency FC of increased (blue) and decreased lineages (red) at 8, 24, 48, and 72hr, and 1wk post-stimulation in IRMs. **(B)** Unique CDR3 number FC of increased (blue) and decreased lineages (red) at 8, 24, 48, and 72hr, and 1wk in IRMs. **(C)** Mutation rate FC of increased (blue) and decreased lineages (red) at 8, 24, 48, and 72hr, and 1wk post-stimulation in IRMs.(Paired Wilcox-ranked test).


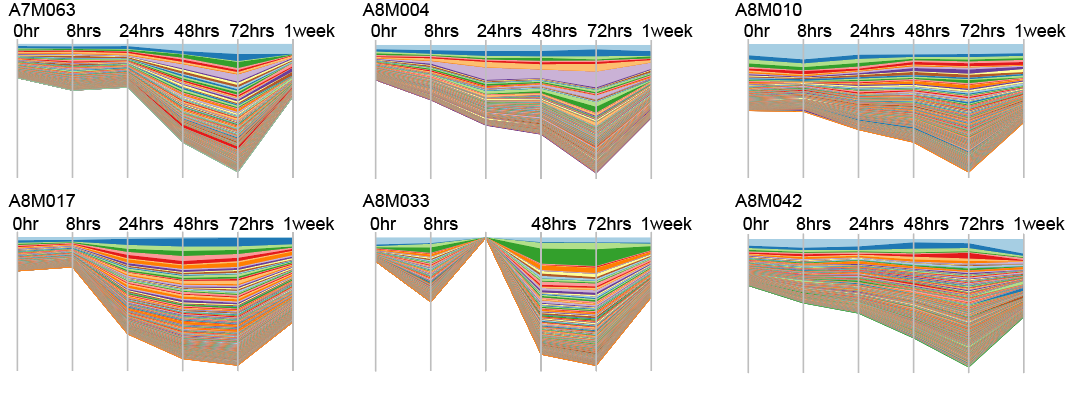


**Figure S16.** **The frequency of expanded-lineages at each time-point in AGMs.** Each bar represents a unique antibody and the width of bar represents the antibody frequency.


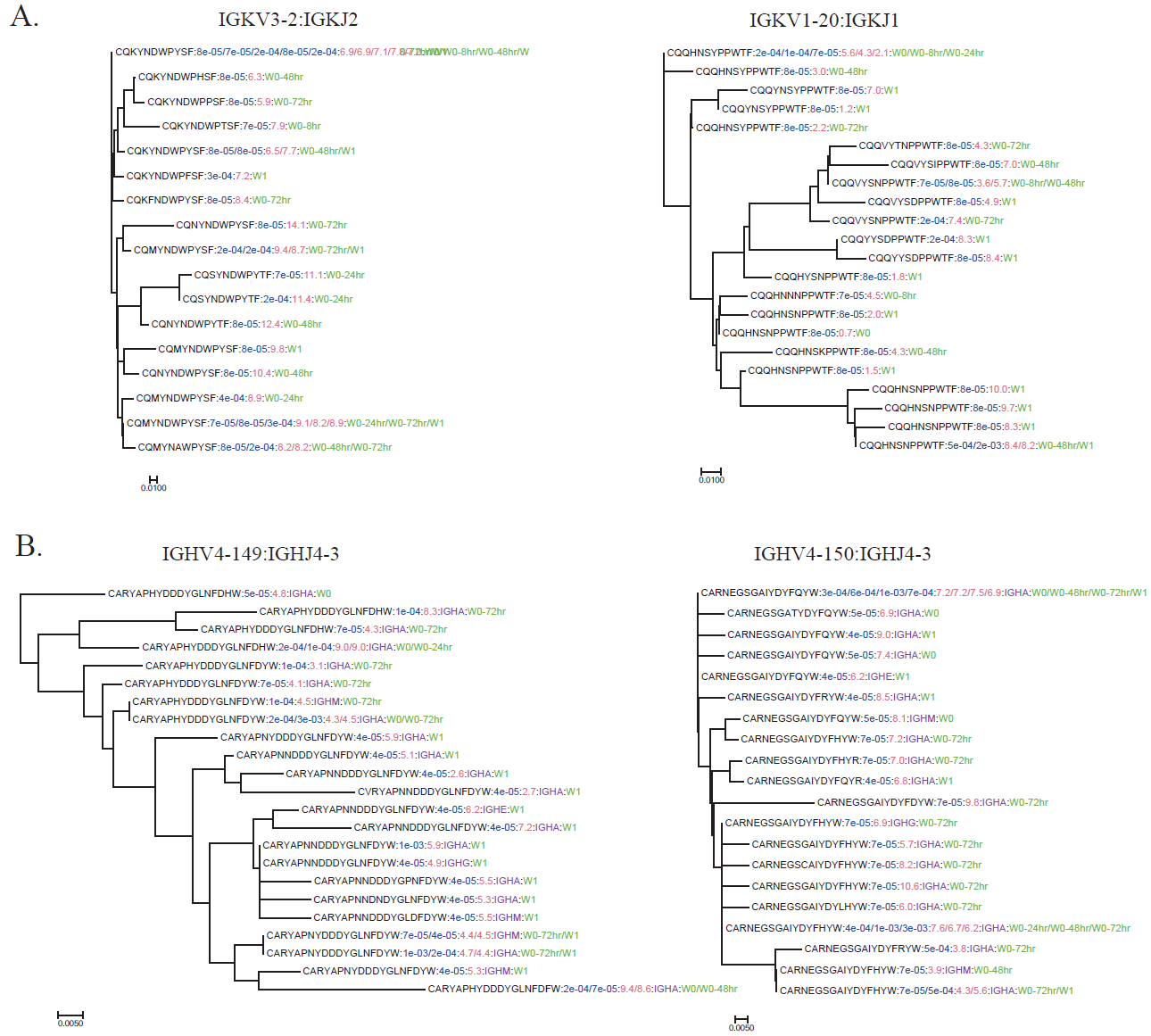


**Figure S17. Phylogenetic tree displayed representative expanded lineages.** (A) two lineages from IgKL repertoire. (B) wo lineages from IgH repertoire. Sequences containing the CDR3 region, J gene region, and a 10-bp non-CDR3 segment of the V gene region were used for construction. All sequences in this lineage belonged to the same V and J genes. Blue text indicates sequence frequency in the sample; red text, sequence mutation rate; purple text, isotype; and green text, samples containing the sequence.


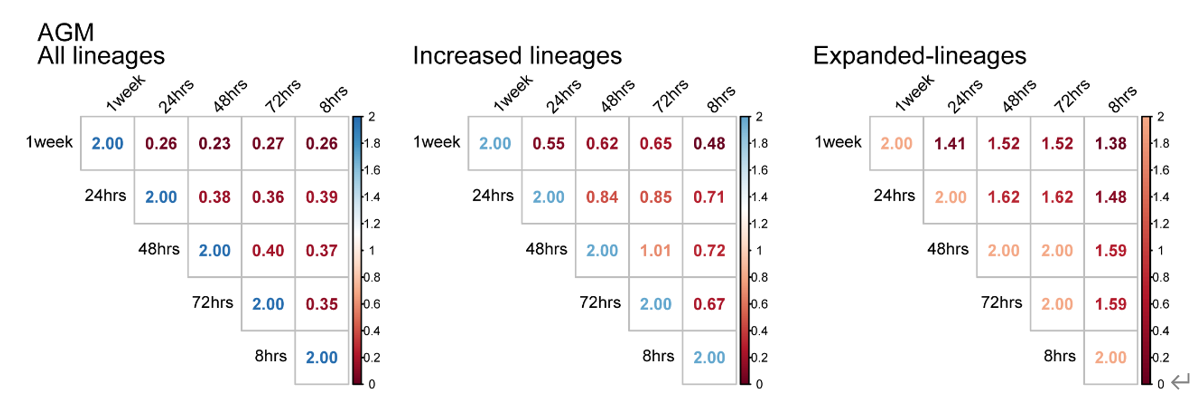


**Figure S18. Statistics of lineage overlapping among timepoints for AGMs.** The overlapping index of all lineages, increased lineages, and expanded among timepoints.


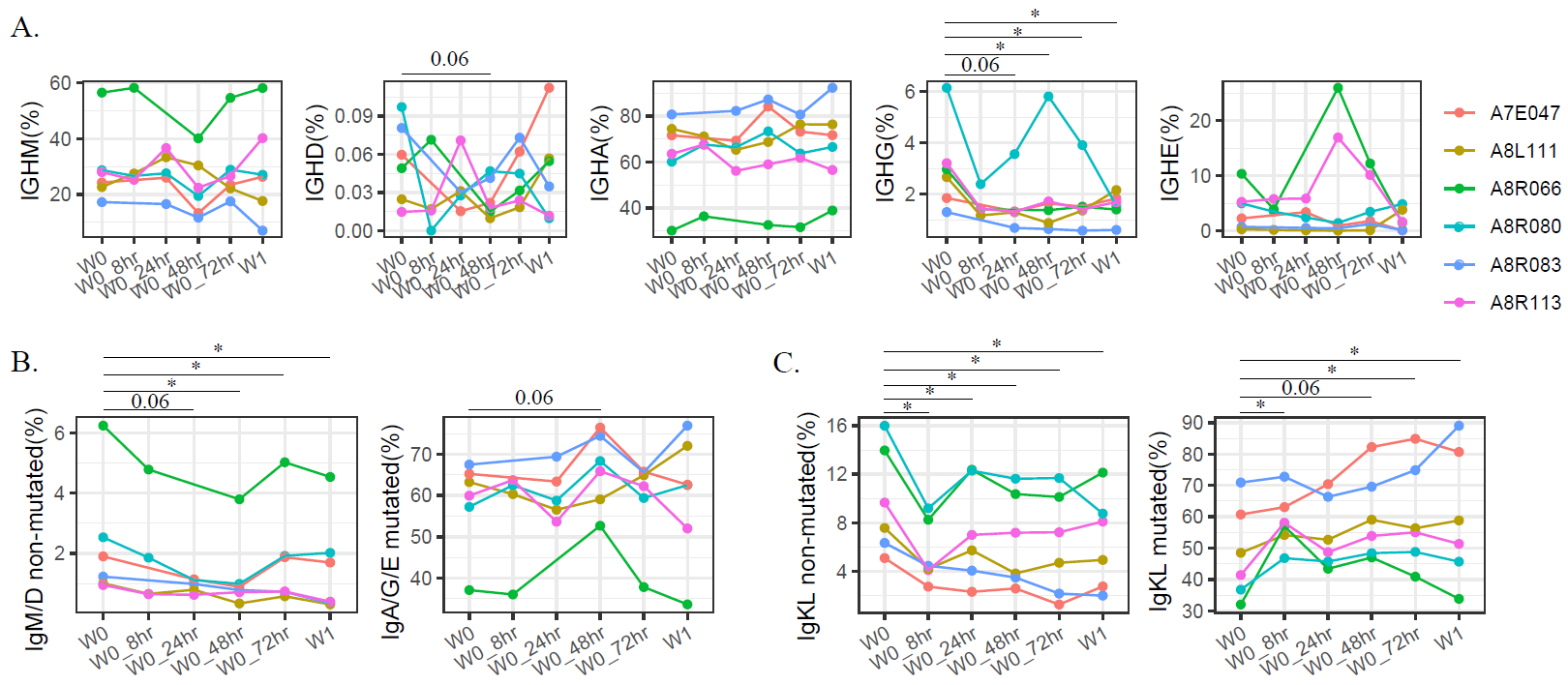


**Figure S19. Proportion of isotypes and mutation distribution in expanded lineages**. (A) Proportion of each isotype in the IgH repertoire across all timepoints. (B) Proportion of IgM/D non-mutated sequences (IgM or IgD with mutation rates < 0.5%) and IgA/G/E mutated sequences (IgA, IgG, or IgE with mutation rates > 3%).(C) Proportion of IgKL non-mutated sequences (mutation rates < 0.5%) and IgKL mutated sequences (mutation rates > 4%).
